# Comparative morphology and evolution of the cnidosac in Cladobranchia (Gastropoda: Heterobranchia: Nudibranchia)

**DOI:** 10.1186/s12983-018-0289-2

**Published:** 2018-11-13

**Authors:** Jessica A. Goodheart, Sabrina Bleidißel, Dorothee Schillo, Ellen E. Strong, Daniel L. Ayres, Angelika Preisfeld, Allen G. Collins, Michael P. Cummings, Heike Wägele

**Affiliations:** 1 Laboratory of Molecular Evolution, Center for Bioinformatics and Computational Biology, University of Maryland, College Park, MD 20742 USA; 2 NMFS, National Systematics Laboratory, National Museum of Natural History, Smithsonian Institution, MRC-153, PO Box 37012, Washington, DC 20013 USA; 3 Department of Invertebrate Zoology, Smithsonian Institution, National Museum of Natural History, MRC 163, P.O. Box 37012, Washington, DC 20013-7012 USA; 4 Present address: Marine Science Institute, University of California Santa Barbara, Santa Barbara, CA 93106 USA; 5 Zoology and Didactics of Biology, University of Wuppertal, 42097 Wuppertal, Germany; 6 Zoologisches Forschungsmuseum Alexander Koenig, 53113 Bonn, Germany

**Keywords:** Nudipleura, Morphological evolution, Nematocyst sequestration, Aeolid, Defense

## Abstract

**Background:**

A number of shelled and shell-less gastropods are known to use multiple defensive mechanisms, including internally generated or externally obtained biochemically active compounds and structures. Within Nudipleura, nudibranchs within Cladobranchia possess such a special defense: the ability to sequester cnidarian nematocysts – small capsules that can inject venom into the tissues of other organisms. This ability is distributed across roughly 600 species within Cladobranchia, and many questions still remain in regard to the comparative morphology and evolution of the cnidosac – the structure that houses sequestered nematocysts (called kleptocnides). In this paper, we describe cnidosac morphology across the main groups of Cladobranchia in which it occurs, and place variation in its structure in a phylogenetic context to better understand the evolution of nematocyst sequestration.

**Results:**

Overall, we find that the length, size and structure of the entrance to the cnidosac varies more than expected based on previous work, as does the structure of the exit, the musculature surrounding the cnidosac, and the position and orientation of the kleptocnides. The sequestration of nematocysts has originated at least twice within Cladobranchia based on the phylogeny presented here using 94 taxa and 409 genes.

**Conclusions:**

The cnidosac is not homologous to cnidosac-like structures found in Hancockiidae. Additionally, the presence of a sac at the distal end of the digestive gland may have originated prior to the sequestration of nematocysts. This study provides a more complete picture of variation in, and evolution of, morphological characters associated with nematocyst sequestration in Cladobranchia.

**Electronic supplementary material:**

The online version of this article (10.1186/s12983-018-0289-2) contains supplementary material, which is available to authorized users.

## Background

A number of shelled and shell-less mollusks are known to use internally generated (endogenous) or externally obtained (exogenous) biochemically active compounds [1, 2] and nematocysts [3, 4], as well as crypsis and aposematism in a defensive capacity [5]. Lineages that are known to possess some of these defenses include the heterobranch groups Sacoglossa (both chemical defenses and crypsis [6]), Anaspidea (chemical and behavioral defenses, e.g., inking [7, 8]), and Nudipleura (aposematism, crypsis and chemical defenses [5, 9, 10]), among others [11–14]. Within Nudipleura, members of a group of nudibranchs called Cladobranchia possess such alternative defenses [4, 15], which have been hypothesized to have contributed to the large-scale diversification of Cladobranchia [4].

In particular, some taxa within Cladobranchia possess the ability to sequester nematocysts from their cnidarian prey. Termed kleptocnides once sequestered, these small venom-filled capsules contain an eversible tubule, often with spines or barbs, that can be discharged into the tissues of other organisms [16, 17] and are used by members of Cnidaria to sting predators and capture food [18]. The sequestration of cnidarian nematocysts occurs primarily in one group of cladobranchs, Aeolidida (commonly referred to as aeolids), which appears to be monophyletic [19]. Additional species within the cladobranch families Hancockiidae and Embletoniidae are also known to sequester nematocysts, but the relationships of these two families to nematocyst sequestering taxa in Aeolidida, and to each other, has long been uncertain [20–22]. A recent phylogenenomic study suggests *Hancockia* to be affiliated with non-aeolid cladobranchs [19]. These results support the hypothesis that nematocyst sequestration has originated at least twice within Cladobranchia [23, 24]. However, representatives of Embletoniidae have not been included in any recent phylogenomic analyses, so its position among cladobranch taxa remains unclear.

**Fig. 1:**
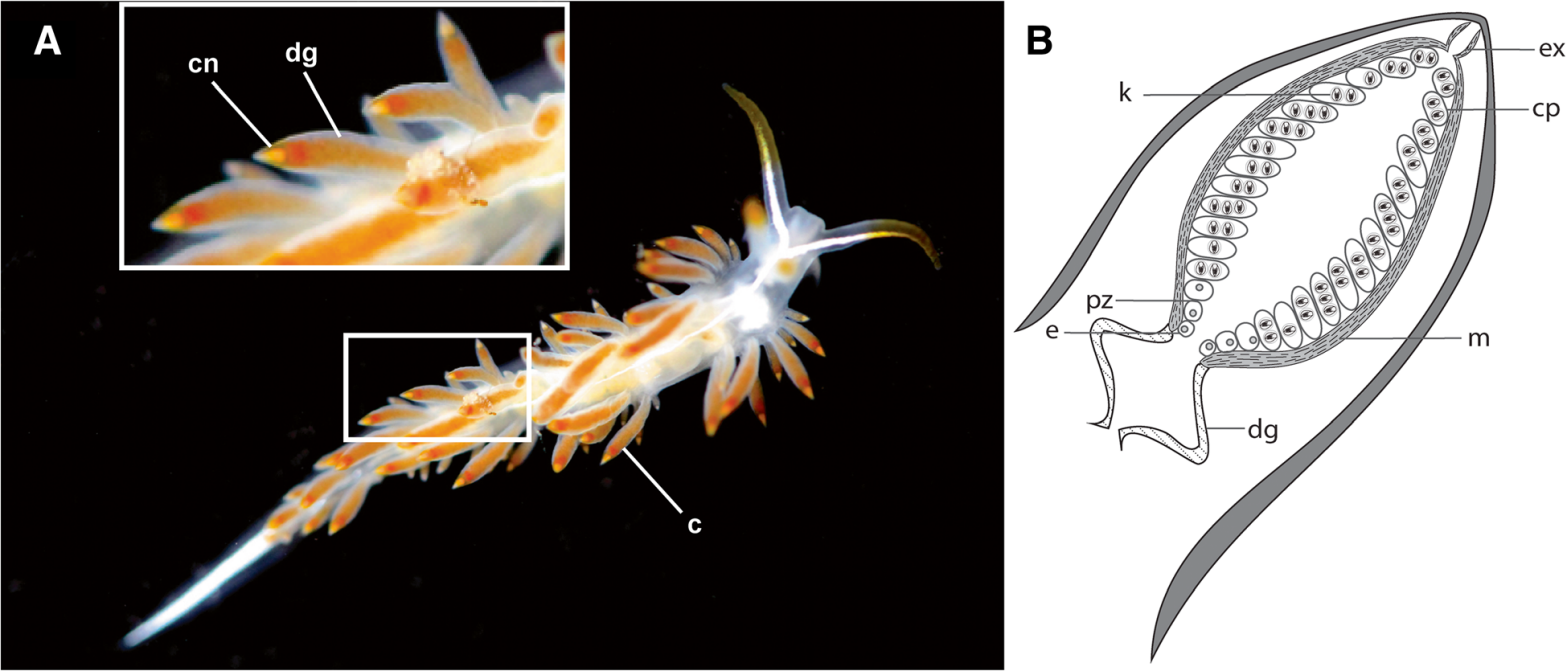
Morphology of the cerata. **a**) Dorsal view of *Orienthella trilineata* (USNM 1408860). Inset: detail of the cerata, and **b**) generalized cnidosac schematic highlighting the main morphological features of the cnidosac. Abbreviations: c, ceras; cn, cnidosac; cp, cnidophage, dg, digestive gland; e, entrance (or ciliated channel in some cases); ex, exit (or pore in some cases); k, kleptocnides; m, musculature; pz, proliferation zone

The process of nematocyst sequestration has been described previously [3, 23], so we will summarize it only briefly here. Nematocysts are a particular type of cnidae, and are complex intracellular organelles housed within cells called cnidocytes. During ingestion of cnidarian tissues, the cnidocyte (the cell) is separated from its nematocyst (the organelle). Nematocysts are then passed through the digestive gland and incorporated into epithelial cells lining a structure called the cnidosac [3, 23], found in aeolids. The cnidosac is a distal extension of the digestive gland within dorsal body outgrowths termed cerata [25] (Fig. 1). The structure is often surrounded by musculature, which contracts to forcibly discharge the sequestered nematocysts through an opening in the tip of each ceras [3, 23]. Structures similar to the cnidosac are present within some members of Hancockiidae, and possibly Embletoniidae [26, 27]. Nematocysts are not functional when taken up by the cnidophages but mature via proton transport within the cnidosac [28].

In many aeolid taxa, descriptions of cnidosac morphology are rare, and when present tend to be vague and unhelpful for detailed analyses. This, coupled with difficulty reconstructing the phylogeny of Aeolidida, and Cladobranchia as a whole, has led to many unanswered questions regarding the comparative morphology and evolution of the cnidosac. For one, it is still unknown whether the cnidosac and similar structures within nematocyst sequestering species from Hancockiidae and Embletoniidae are homologous [26, 27, 29–32]. Based on recent phylogenomic work, we hypothesize that the similar structure present in Hancockiidae is not homologous to the cnidosac, but the homology of the structure in Embletoniidae remains uncertain. Further, the incorporation of an organelle that can be subsequently used by the sequestering animal allows for the hypothesis that the structures associated with this ability may be specific to particular types of organelles. Since there is diversity in nematocyst morphology among the cnidarian prey of sequestering taxa [33–37], we hypothesize that cladobranchs may have evolved specialized cnidosac structures to maneuver and arrange a variety of nematocyst types for storage. The transition to non-cnidarian prey types may have also led to the loss of functional cnidosac features in some taxa.

In this paper, we describe cnidosac morphology across the main groups of Cladobranchia in which it occurs and discuss possible functions for the variation in structural characters in a phylogenetic context. We then combine these morphological data with a phylogeny for Cladobranchia inferred using molecular data (integrating RNA-Seq and PCR-based sequencing data), which allows for a more complete picture of the evolution of nematocyst sequestration within this group.

## Methods

### Morphological data collection

Individuals were relaxed in 10% magnesium chloride when possible, followed by fixation in 10% Bouin’s solution or ~ 4–6% saltwater formalin. For plastic sectioning, whole animals were dehydrated in ethanol and embedded in Hydroxypropyl methacrylate [38]. Serial sections (2.5 μm) were stained with Toluidine blue for 15–20 s (for the majority of specimens), which stains neutral mucopolysaccharides, nucleic acids and proteins shades of blue, and acidic mucopolysaccharides red to violet. For paraffin sectioning, individual cerata were dehydrated in ethanol and embedded in paraffin. Serial sections of 6 μm were made and stained with a modified Masson’s trichrome stain [39]. Information on the histological slides used in our analyses is provided in Additional file 1. For many species, only one individual was available for histological analysis, but at least two, and up to ten, cerata were observed to account for variation in structure and contents across the body axes of individual animals (anterior-posterior and medial-lateral). Additional data were collected from the literature [15, 26, 27, 40–45].

### Taxon sampling

Molecular data were collected for a total of 90 cladobranchs and four outgroup taxa (Additional file 2). The majority of these taxa are from Aeolidida, with some taxa from the other major clades of Cladobranchia to assess nematocyst sequestration evolution across this group. These molecular data include RNA-Seq data for 40 taxa taken from the NCBI Sequence Read Archive [46], along with additional PCR-based Sanger sequencing data from GenBank [47] (11 taxa) and 43 newly sequenced individuals.

### Molecular data collection – PCR-based

Specimens were fixed in 96% ethyl alcohol and stored partly at room temperature or in a refrigerator at ~ 7 °C. DNA isolation was carried out by means of *DNeasy Blood and Tissue-Kit* (QIAgen®), DNeasy Plant Mini Kit (QIAgen®), or E.Z.N.A. Invertebrate DNA Kit (Peqlab), following the manufacturer protocols. Under sterile conditions slices of the foot or preferably dorsal tissue of approximately 5 mm^2^ were taken and ground with a pestle. Proteinase K was added to assist with lysis. To ensure efficient lysis, the samples were placed in a 56 °C shaking bath and lysed over night. The contents of the reaction tube were then transferred to a silica-membrane mini spin-column with collection tube and centrifuged. Two washing steps were performed to eliminate the remaining contaminants and enzyme inhibitors. The purified DNA was then eluted in two successive steps using 50 μL of low-salt buffer each. The extracted DNA was then stored at − 20 °C.

For some PCR reactions the QIAGEN® Multiplex PCR Kit was used according to manufacturer instructions. Each PCR reaction used 2.3 μL RNAse-free H_2_O, 2.0 μL 5x Q-Solution, 10.0 μL 1x QIAGEN® Multiplex PCR Master Mix, and 1.6 μL of each primer at a concentration of 10 pmol/μL. The primers used for each gene fragment are listed in Additional file 3. The thermocycler parameters for the PCR reactions for each gene are presented in Additional file 4. In some cases, as indicated in Additional file 4, a touchdown PCR protocol was used to ensure enrichment of the correct product and minimize non-specific binding. The QIAquick™ PCR Purification Kit, ExoSAP-IT™, or E.Z.N.A. Cycle-Pure Kit (Omega) were then used for PCR product purification. Bi-directional sequencing was completed by IIT Biotech/Bioservice, Bielefeld or Eurofins MWG Operon.

### Extraction of sequences from transcriptome data

To extract sequences for the mitochondrial genes Cytochrome Oxidase I (COI) and 16S rRNA, and the nuclear gene 18S rRNA sequences from each transcriptome, the datasets were first used to create BLAST databases using makeblastdb from the BLAST [48] command line application. Sequences from the most closely related organisms in GenBank [47] were then aligned to the transcriptome databases using tblastn (COI) or blastn (16S and 18S). The top hit with the lowest e-value and the longest sequences were then selected from the hits. These were then manually trimmed to match the most common sequence lengths for each gene.

### Alignments and construction of sequence matrix

Sequences from each gene (COI, 16S, and 18S) were aligned using MAFFT version 7.187 [49] using the --auto option. The individual gene alignments were then concatenated with the *nt123* matrix from Goodheart et al. [19]. Sites not represented by sequence data in at least four taxa were removed from the matrix. The final alignment contained 94 taxa, 409 genes, and 610,169 sites.

### Phylogenetic analysis

Phylogenetic analysis included the following partitioning scheme: 1) protein coding genes were partitioned by codon position, and 2) the two rRNA genes (16S and 18S) were partitioned by gene. To conduct the phylogenetic analysis we used RAxML (v.8.2.9; [50]) using both the CIPRES Science Gateway [51] and Extreme Science and Engineering Discovery Environment (XSEDE) [52]. We used the default settings in RAxML, with a general time reversible substitution model (GTR; [53]) with a rate heterogeneity model with a gamma distribution (+G; [54]) for each partition. Five best tree searches were conducted and the tree with the highest likelihood was considered the most optimal, and 500 bootstrap replicates were completed. Gene tree analyses for each of the three added genes and a concatenated 3 genes analysis were completed in a modified version of MrBayes (v3.2.6; [55]), with a prerelease version of BEAGLE library [56] to make use of highly-parallel processors to speed up the core calculations for the phylogenetic analysis. The version of the BEAGLE library supports heterogeneous hardware [57], used for analyses here on both CPUs and GPUs, and algorithms to improve performance for partitioned analyses and independent subtrees [58]. We used a general time reversible substitution model (GTR; [53]) with a rate heterogeneity model with a proportion of invariant sites estimated (+I; [59]) and the remainder with a gamma distribution (+G; [54]). The analysis was run for 10 million generations and sampled every 1000 generations, and the first 25% of trees were discarded as burn-in. MrBayes default settings were retained for the rest of the analysis parameters, including construction of the consensus tree. Convergence was assessed in R [60] using the RWTY package [61] (Additional files 5, 6, 7, 8). We attempted to run the full analysis on the same platform as the gene trees, but none would converge.

### Ancestral state reconstruction

Ancestral states were reconstructed for two characters: (i) the presence or absence of a sac (that we define as a bag-like structure composed of several cells) at the distal edge of the digestive gland (i.e., a distal sac), and (ii) the presence or absence of kleptocnides. Using these character states, we compared the fit of three discrete trait models using the corrected AIC (AICc; corrected for small sample sizes) from the AICcmodavg 2.0–4 package [62] in R 3.3.1 [60]. We assessed fit for two models, where: (i) all transition rates were equal (ER; same as the symmetrical model in this case); (ii) forward and reverse transitions were different between states (all rates different, ARD). The ARD model (kleptocnides AICc = 67.62823; sac AICc = 47.02553) was a slightly better fit to the data than the ER model (kleptocnides AICc = 67.63039; sac AICc = 49.98232) for each character. The final ancestral state reconstruction analysis was completed using the ace function, in the APE package [63], under the ARD model using default parameters. The ace function uses a Markov model employing a maximum likelihood approach. In this analysis, the marginal ancestral states are returned, which are given as the proportion of the total likelihood calculated for each state at each node.

## Results

### Cnidosac terminology

In this paper, we clarify the terminology and define how we use each term in order to prevent ambiguity and to encourage consistency in descriptions of the cnidosac in the future. A summary of the terminology as used here and how it relates to that in previous publications is provided in Table 1. The term cnidosac (to describe the structure that houses kleptocnides in the tip of the ceras) has been in use for over 100 years, but terminology to describe many aspects of the cnidosac and related structures has been inconsistent [3, 31, 64–69]. Our use of the terms nematocyst and kleptocnide is particularly deliberate. No study exists that clearly identifies the types of cnidocysts that may be incorporated into the cnidosac, and it is impossible to say with absolute certainty whether only nematocysts, or other types of cnidocysts, such as spirocysts or ptychocysts, may also be sequestered. This is largely due to the fact that researchers have previously identified kleptocnides based on the definition that nematocysts have a tubule wrapped around a shaft, which may also be used to describe spirocysts [70]. As such, use of the term “nematocyst” is more precise, but might be incorrect. However, both additional types of cnidae are present only in Hexacorallia (Anthozoa), and so would not be found in any nudibranch that feeds on other types of cnidarian prey (the majority of the taxa within sequestering groups). Further, neither spirocysts nor ptychocysts are known to contain venom and both are presumed to have an adhesive function [37], which would make them non-functional in a defensive capacity. With these factors in mind, we choose to use the term nematocyst to refer to the structures stolen from cnidarians. For clarity, the term kleptocnide refers to these structures after they have been sequestered within the cnidosac (or similar structure) of the nudibranchs. For example, not all nematocysts found within the cnidarian prey species, or within the digestive tract of the nudibranchs, will necessarily become kleptocnides.

**Table 1:** Equivalency table for terminology related to the cnidosac from published research [3, 30, 31, 64–69, 74]

Author Herein	Cnidosac	Cnidocysts	Channel	Exit/Cnidopore	Cnidophage	Digestive gland
Hancock & Embleton 1845	ovate vesicle/sac	elliptical bodies with slender, hair-like filaments	ciliated channel	external orifice	little transparent ellipsoidal membranous bags	liver caecum
Wright 1863	ovoid vesicle/sac	cnidae, thread cells	–	–	–	biliary sac
Herdman & Clubb 1889	cnidophorous sac	cnida	connecting tube, ciliated canal	terminal opening	cnidocyst	hepatic caecum
Herdman 1890	cnidophorous sac	cnida, thread cells	slender tube with thin walls & few muscle fibers	clearly defined aperature at the apex	cnidocyst	hepatic diverticulum
Grosvenor 1903	cnidophorous sac/cnidosac/cnidophore	nematocysts	ciliated canal	terminal opening	cnidocyst/cnidoblast	alimentary canal/ diverticula of the gastric gland
Edmunds 1966	cnidosac	nematocysts	–	cnidopore	cells	digestive gland
Kälker & Schmekel 1976	cnidosac	cnidocysts	pore/ narrow duct	entrance	nematophage	hepatopancreas
Conklin & Mariscal, 1977	cnidosac	nematocysts	–	cnidopore	cnidocyst	
Ohkawa & Yamasu, 1993	cnidosac	nematocysts	–	–	cnidophage cell	digestive diverticulum
Greenwood 2009	cnidosac	nematocysts/kleptocnidae	–	–	cnidophage cell	digestive diverticula

The term cnidosac-like refers only to structures similar to the cnidosac found within taxa that are not aeolids. It is a broad term only intended to indicate the lack of homology with the cnidosac itself.

### General structure of cnidosacs

**Fig. 2:**
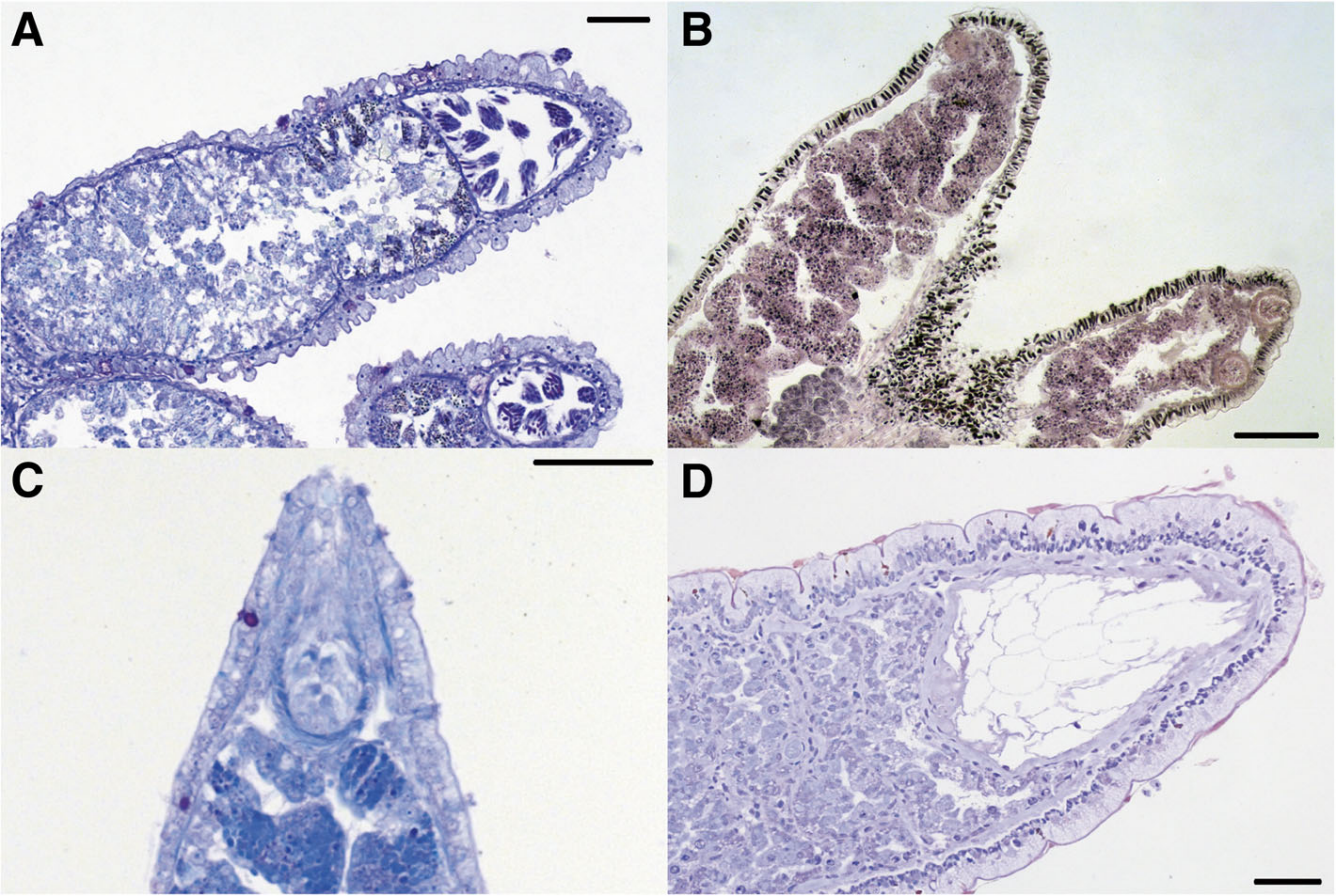
Variation in number of cnidosacs and presence/absence of kleptocnides: **a**) longitudinal section of a single cnidosac in one ceras from *Caloria elegans* (scale bar = 50 μm); **b**) longitudinal section of multiple cnidosacs in the dendronotid *Hancockia californica* (scale bar = 100 μm); and **c**-**d**) longitudinal sections of cnidosacs lacking kleptocnides: **c**) *Favorinus auritulus* (USNM1276034), and **d**) *Phyllodesmium colemani*. Scale bars = 50 μm

The cnidosac is a muscular prolongation of the digestive gland located in the apex of each ceras within members of the nudibranch group Aeolidida (Tables 2 and 3). Similar structures found within the family Hancockiidae are here considered cnidosac-like structures, due primarily to an independent origin of nematocyst sequestration (see ancestral state reconstruction results). The cnidosac connects to the digestive gland via a single entrance, which in many cases is directly adjacent to a zone of epithelial cell proliferation that moves cells distally to line the inside of the cnidosac. A constriction of the musculature is present near the tip of the cnidosac in some taxa, just before an exit from the cnidosac to the external environment. There is a single cnidosac per ceras in the case of aeolids, and multiple cnidosac-like structures per ceras in species of *Hancockia* (Fig. 2b). The cnidosac, as defined here, is exclusively found within members of Aeolidida, but not all species that possess cnidosacs sequester nematocysts. For example, kleptocnides are not present in members of the genus *Favorinus* and most species within *Phyllodesmium* (Fig. 2c, d)*.*

**Table 2:** Morphological data on distal sacs and the presence of kleptocnides for all species evaluated in this study

Family	Species	Distal sac	Kleptocnides	Reference
Lomanotidae	*Lomanotus vermiformis*	Absent	Absent	[15]
Dotidae	*Doto lancei*	Absent	Absent	[15]
Bornellidae	*Bornella anguilla*	Absent	Absent	[15]
Dendronotidae	*Dendronotus venustus*	Absent	Absent	[15]
Scyllaeidae	*Scyllaea fulva*	Absent	Absent	This study
Tethyidae	*Melibe leonina*	Absent	Absent	[42]
Arminidae	*Armina californica*	Absent	Absent	[15]
	*Dermatobranchus sp.*	Absent	Absent	This study
Tritoniidae	*Tritonia hamnerorum*	Absent	Absent	[15]
	*Tritoniopsis frydis*	Absent	Absent	[15]
	*Tritonia festiva*	Absent	Absent	[15]
	*Tritonia diomedea*	Absent	Absent	[15]
Charcotiidae	*Charcotia granulosa*	Present	Absent	[43]
	*Pseudotritonia gracilidens*	Present	Absent	[41]
	*Pseudotritonia telarma*	Present	Absent	[15]
Dironidae	*Dirona albolineata*	Absent	Absent	[44]
	*Dirona picta*	Absent	Absent	This study
Proctonotidae	*Janolus barbarensis*	Absent	Absent	This study
	*Janolus capensis*	Present	Absent	This study
	*Janolus cristatus*	Present	Absent	This study
Hancockiidae	*Hancockia uncinata*	Present	Present	[27]
Aeolidiidae	*Aeolidia papillosa*	Present	Present	This study
	*Bulbaeolidia alba*	Present	Absent	This study
	*Anteaeolidiella chromosoma*	Present	Present	This study
	*Berghia stephanieae*	Present	Present	This study
	*Cerberilla amboinensis*	Present	Present	This study
	*Limenandra confusa*	Present	Present	This study
	*Spurilla neapolitana*	Present	Present	This study
Facelinidae 1	*Austraeolis stearnsi*	Present	Present	This study
	*Caloria elegans*	Present	Present	This study
	*Cratena peregrina*	Present	Present	This study
	*Facelina rubrovittata*	Present	Present	This study
	*Favorinus auritulus*	Present	Absent	This study
	*Glaucus atlanticus*	Present	Present	This study
	*Learchis evelinae*	Present	Present	This study
	*Palisa papillata*	Present	Present	This study
	*Phidiana lottini*	Present	Present	This study
	*Phidiana lynceus*	Present	Present	This study
	*Pruvotfolia pselliotes*	Present	Present	This study
	*Pteraeolidia ianthina*	Present	Present	This study
Facelinidae 2	*Dondice occidentalis*	Present	Present	This study
	*Hermissenda crassicornis*	Present	Present	This study
	*Hermissenda opalescens*	Present	Present	This study
	*Noumeaella rubrofasciata*	Present	Present	This study
	*Phyllodesmium* cf. *magnum*	Present	Absent	This study
	*Phyllodesmium colemani*	Present	Absent	This study
	*Phyllodesmium jakobsenae*	Present	Present	This study
	*Phyllodesmium koehleri*	Present	Absent	This study
	*Phyllodesmium macphersonae*	Present	Absent	This study
Paracoryphellidae	*Ziminella salmonacea*	Present	Present	This study
Coryphellidae	*Itaxia falklandica*	Present	Absent	This study
	*Microchlamylla gracilis*	Present	Present	This study
Unidentiidae	*Unidentia angelvaldesi*	Present	Present	[40]
Embletoniidae	*Embletonia gracilis*	Present	Present	This study
	*Embletonia pulchra*	Present	Present	[26]
Fionidae	*Cuthona albocrusta*	Present	Present	This study
	*Cuthona caerulea*	Present	Present	This study
	*Cuthona kanga*	Present	Present	This study
	*Fiona pinnata*	Absent	Absent	This study
	*Phestilla sp.*	Present	Absent	This study
	*Tergipes antarcticus*	Absent	Absent	This study
	*Tergipes tergipes*	Absent	Absent	This study
Flabellinidae	*Calmella cavolini*	Present	Present	This study
	*Coryphellina rubrolineata*	Present	Present	[45]
	*Edmundsella pedata*	Present	Present	This study
	*Flabellina affinis*	Present	Present	This study
	*Paraflabellina ischitana*	Present	Present	This study
	*Paraflabellina gabinierei*	Present	Present	This study
Samlidae	*Luisella babai*	Present	Present	This study
Flabellinopsidae	*Flabellinopsis iodinea*	Present	Present	This study
Notaeolidiidae	*Notaeolidia depressa*	Present	Present	This study

**Table 3:** Morphological data on the cnidosac and cnidosac-like structures of nematocyst sequestering species evaluated in this study

Species	Musculature	Proliferation zone	Entrance/Channel	Exit	Constriction near the tip of the cnidosac
*Hancockia uncinata*	multi-layered	No	Entrance	Exit	Absent
*Hancockia schoeferti*	thick	No	Entrance	Exit	Absent
*Hancockia californica*	multi-layered	No	Entrance	Exit	Absent
*Aeolidia papillosa*	multi-layered	Yes	Channel	Pore	Absent
*Bulbaeolidia alba*	Absent	No	Unobserved	Unobserved	Absent
*Anteaeolidiella chromosoma*	multi-layered	Yes	Inferred	Pore	Absent
*Berghia stephanieae*	multi-layered	Yes	Inferred	Unobserved	Absent
*Cerberilla amboinensis*	multi-layered	Yes	Channel	Pore	Present
*Limenandra confusa*	multi-layered	Yes	Inferred	Exit	Present
*Spurilla neapolitana*	multi-layered	Yes	Entrance	Unobserved	Absent
*Austraeolis stearnsi*	single layer	No	Unobserved	Pore	Present
*Caloria elegans*	two layers	Yes	Unobserved	Unobserved	Absent
*Cratena peregrina*	multi-layered	Yes	Channel	Exit	Absent
*Facelina rubrovittata*	multi-layered	No	Unobserved	Exit	Present
*Favorinus auritulus*	multi-layered	Yes	Unobserved	Exit	Absent
*Glaucus atlanticus*	multi-layered	Yes	Unobserved	Unobserved	Inconclusive
*Learchis evelinae*	multi-layered	Yes	Entrance	Exit	Absent
*Palisa papillata*	multi-layered	Inconclusive	Unobserved	Exit	Inconclusive
*Phidiana lottini*	multi-layered	No	Unobserved	Exit	Inconclusive
*Phidiana lynceus*	multi-layered	Yes	Unobserved	Exit	Present
*Pruvotfolia pselliotes*	multi-layered	Yes	Unobserved	Exit	Absent
*Pteraeolidia ianthina*	multi-layered	Yes	Channel	Exit	Absent
*Dondice occidentalis*	single layer	Yes	Entrance	Unobserved	Absent
*Hermissenda crassicornis*	multi-layered	Inconclusive	Entrance	Exit	Absent
*Noumeaella* sp.	multi-layered	Yes	Entrance	Exit	Absent
*Phyllodesmium* cf. *magnum*	single layer	No	Unobserved	Unobserved	Absent
*Phyllodesmium colemani*	single layer	No	Unobserved	Unobserved	Absent
*Phyllodesmium jakobsenae*	single layer	Yes	Entrance	Unobserved	Absent
*Phyllodesmium koehleri*	single layer	No	Unobserved	Unobserved	Absent
*Phyllodesmium macphersonae*	single layer	No	Entrance	Unobserved	Absent
*Ziminella salmonacea*	multi-layered	Yes	Channel	Unobserved	Absent
*Itaxia falklandica*	multi-layered	Inconclusive	Unobserved	Unobserved	Absent
*Microchlamylla gracilis*	single layer	Inconclusive	Unobserved	Unobserved	Absent
*Embletonia gracilis*	Absent	No	Unobserved	Unobserved	Absent
*Embletonia pulchra*	Absent	No	Unobserved	Unobserved	Absent
*Cuthona albocrusta*	single layer	Yes	Entrance	Unobserved	Absent
*Cuthona caerulea*	single layer	Inconclusive	Entrance	Exit	Absent
*Cuthona kanga*	multi-layered	Yes	Unobserved	Exit	Absent
*Fiona pinnata*	–	–	–	–	–
Phestilla sp.	multi-layered	Inconclusive	Entrance	Exit	Absent
*Tergipes antarcticus*	–	–	–	–	–
*Tergipes tergipes*	–	–	–	–	–
*Calmella cavolini*	single layer	Yes	Entrance	Exit	Absent
*Edmundsella pedata*	multi-layered	Yes	Entrance	Unobserved	Absent
*Flabellina affinis*	multi-layered	Yes	Entrance	Unobserved	Absent
*Paraflabellina ischitana*	multi-layered	Yes	Entrance	Exit	Absent
*Paraflabellina gabinierei*	multi-layered	No	Entrance	Unobserved	Absent
*Luisella babai*	multi-layered	No	Unobserved	Unobserved	Absent
*Flabellinopsis iodinea*	multi-layered	Yes	Entrance	Exit	Absent
*Notaeolidia depressa*	single layer	No	Entrance	Exit	Absent

**Fig. 3:**
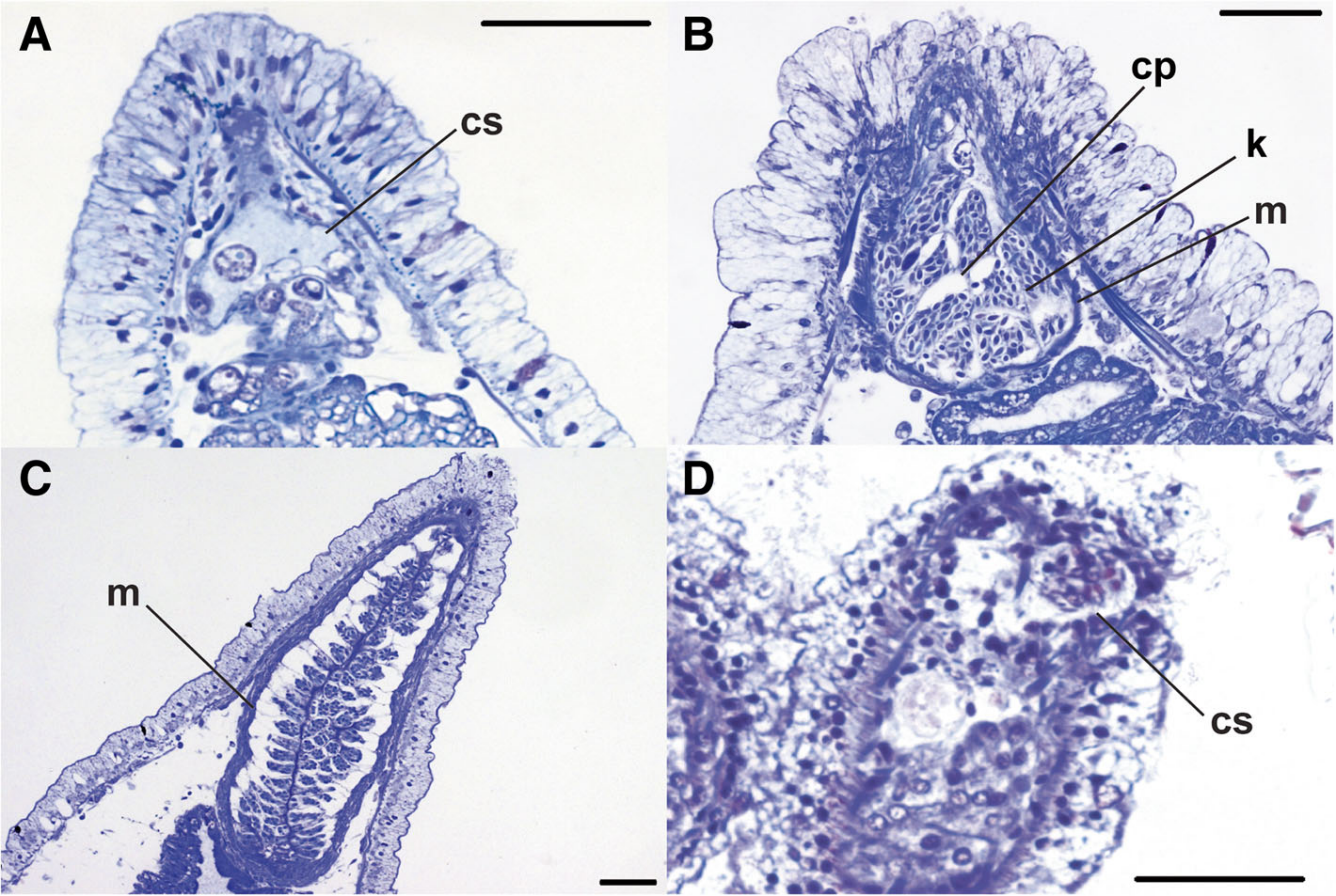
Variation in cnidosac musculature: **a**) longitudinal section showing absence of cnidosac musculature in *Bulbaeolidia alba*; **b**) longitudinal section showing a single muscle layer in *Microchlamylla gracilis*; **c**) longitudinal section showing multi-layered musculature in *Flabellinia affinis*; and **d**) longitudinal section showing the cnidosac in *Embletonia gracilis*. Abbreviations: cs, cnidosac; cp, cnidophage; k, kleptocnides; m, musculature. Scale bars = 50 μm

**Fig. 4:**
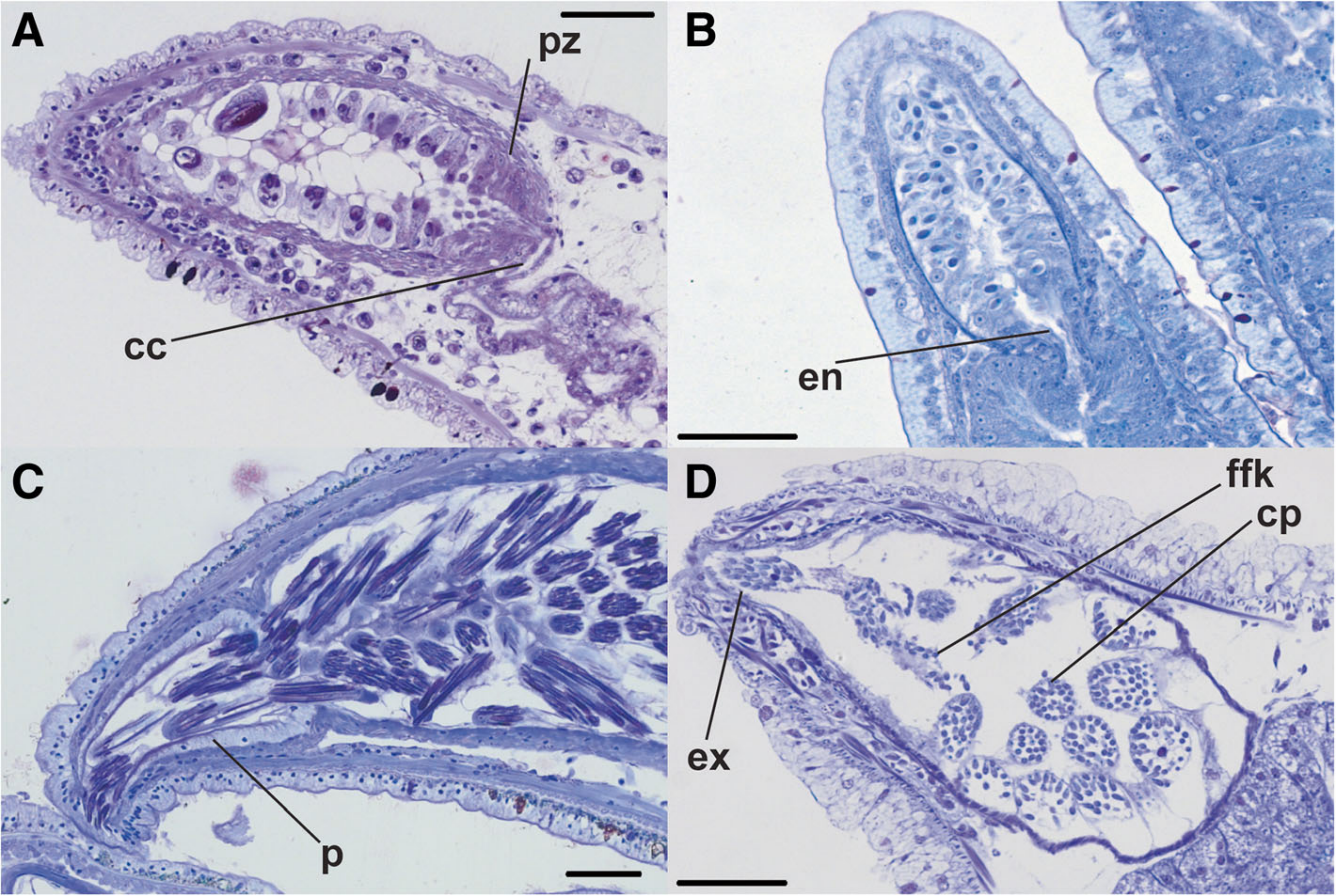
Variation in cnidosac entrance and exit morphology: **a**) longitudinal section showing a ciliated channel in *Pteraeolidia ianthina* (scale bar = 50 μm); **b**) longitudinal section showing a simple entrance from *Dondice occidentalis* (USNM1276036; scale bar = 50 μm); **c**) longitudinal section showing a discrete cnidopore in *Cerberilla amboinensis* (scale bar = 100 μm); and **d**) longitudinal section showing a simple exit in *Cratena peregrina* (scale bar = 50 μm). Abbreviations: cc, ciliated channel; cp, cnidophage; en, entrance; ex, exit; ffk, free-floating kleptocnide; p, cnidopore; pz, proliferation zone

The cnidosac may contain musculature in some taxa, which can vary in the number of layers (Fig. 3b-c). Other taxa do not possess musculature around the cnidosac (Fig. 3a, d). The epithelium of the cnidosac appears to consist exclusively of cells called cnidophages, which may be differentiated according to their position within the cnidosac, from proximal (close to the entrance from the digestive gland) to distal. Close to the entrance of the cnidosac these cells usually still show distinct nuclei, but the appearance of the cells becomes more atypical in the distal part of the cnidosac (Fig. 4b). In particular, cnidophages appear to have no discernable cytoplasm and a pyknotic nucleus towards the distal end of the cnidosac, likely owing to the incorporation of kleptocnides. It is unclear whether the nuclei are simply obscured by the kleptocnide contents or lost through the process of incorporating the kleptocnides. We did not observe an intact epithelial lining within the cnidosac in all species investigated, and this may be due in part to artifacts in the preservation process that caused destruction of the cell membranes, possibly related to the large size of some individuals. In these cases, the surrounding musculature or connective tissue was observed with few or no epithelial cells found. Kleptocnides are usually located within vacuoles inside the cnidophages, and the number of kleptocnides may vary within a vacuole.

The entrance to the cnidosac from the digestive gland may be a broad, open, and simple entrance, such as that found in *Dondice occidentalis* (Fig. 4b), or form a channel of varying length lined by ciliated or cuboidal (i.e., non-digestive) cells (e.g., *Pteraeolidia ianthina*; Fig. 4a). The entrance may be too small to be captured in an individual section in some taxa, and must be inferred based on changes in orientation of cells in the vicinity of the transition between the digestive gland and the cnidosac, as indicated in Table 2. It is also possible that the entrance may be temporary in some taxa. Adjacent to the entrance at the base of the cnidosac is a proliferation zone, where small cells from the proximal cnidosac epithelium seem to form and grow larger as they migrate toward the distal end, likely to accommodate maturing kleptocnides (Fig. 4a). In some cases the contents of the cnidosac may obscure the proliferation zone in sections, making it unclear whether the zone is not present or is simply unobservable. This is true for species within Aeolidida as well as those distantly related to Aeolidida with cnidosac-like structures (e.g., *Hancockia* spp.; Fig. 2b). In other cases, cnidophages, and sometimes the cnidosac as a whole, appear partly, or completely, empty (e.g., *Phyllodesmium colemani* Fig. 2d and *Cratena peregrina*, Fig. 4d). A simple exit from the cnidosac was found in many individuals, covered in some cases with a thin epithelial lining that appears to be associated with the outer epidermis. We refer to a distinct, lasting exit from the cnidosac, in the sense that the epithelium of the cnidosac connects to that of the epidermis, as a cnidopore (Fig. 4c). If this structure is not distinguishable (e.g., the epidermis of the ceras shows only small cuboidal cells at the tip and the epithelium does not connect to the epidermis), we refer to this simply as an exit (Fig. 4d).

However, a few taxa that we present possess variations on the general scheme outlined above, including *Embletonia* spp. (Embletoniidae), *Bulbaeolidia alba* (Aeolidiidae), *Favorinus auritulus* (Facelinidae 1), and *Phyllodesmium* spp. (Facelinidae 2). In *B. alba*, *F. auritulus*, and the majority of *Phyllodesmium* species (except *P. jakobsenae*), a cnidosac was found to be present but no kleptocnides were observed (e.g., Fig. 2d). The structure of the cnidosac in several of these taxa was further observed to present several unique differences compared to those species that harbor kleptocnides. For example, in *Phyllodesmium*, the cnidosac closely resembles those of other aeolids, but typically has only a single layer of musculature, no obvious proliferation zone, and in most cases no exit was observed. *Favorinus auritulus* on the other hand has a multi-layered musculature, an exit, and appears to possess a proliferation zone similar to species that have kleptocnides. Instead of a muscular cnidosac, *Bulbaeolidia alba* possesses a membrane-bound sac at the tip of the ceras which lacks an exit or cnidopore (Fig. 3a). This sac attaches to the digestive gland and contains only zooxanthellae. In *Embletonia gracilis*, the cnidosac lacks musculature and possesses no apparent connections with the digestive gland or external environment (Fig. 3d).

In some observed taxa from Fionidae (*F. pinnata*, *Tergipes tergipes* and *T. antarcticus*)*,* a cnidosac was not present.

### Phylogenetic results

**Fig. 5:**
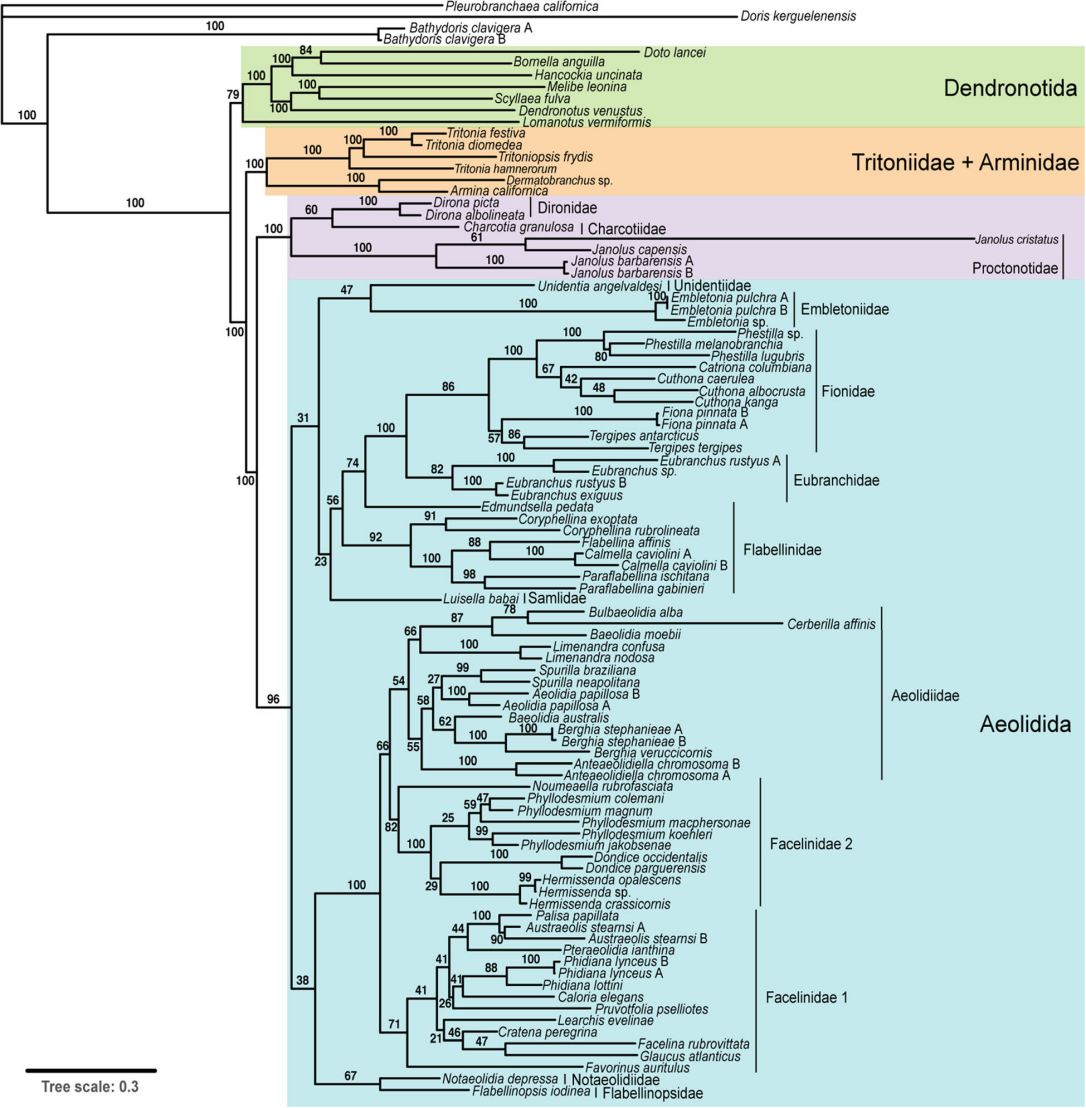
Maximum likelihood phylogeny of Cladobranchia using the taxa and genes presented in Additional file 1. Bootstrap support values are presented adjacent to the corresponding branch

The maximum likelihood phylogeny inferred in this study has varied bootstrap support (BS) among its branches (Fig. 5). Support for Cladobranchia (BS =100), Tritoniidae + Arminidae (BS = 100), Dironidae + Charcotiidae + Proctonotidae (BS = 100), and Aeolidida (BS = 96) is high, but support for Dendronotida (BS = 79) is lower. Bootstrap support values throughout the rest of the tree range from 21 to 100.

Convergence statistics for each gene tree inference (and that of the three genes combined) supported convergence of each analysis by multiple measures (Additional files 5, 6, 7, 8). The average approximate topology effective sample size (ESS) were COI: 1139 and 975, (chain 1 and chain 2 respectively); 16S: 598 and 546; 18S: 6707 and 11,039; and for 3 genes: 8358 and 8597. The tree topology trace shows well-mixed chains, fairly stable cumulative split frequencies, and sliding window split frequencies with large jumps with an apparent extensive exploration of the tree space. The average standard deviation of split frequencies (ASDSF) is below 0.01 and shows a consistent decrease for each analysis, as expected with convergence. The topology of these trees is often consistent with the full phylogeny at the genus (and sometimes family) level, but largely inconsistent at deeper nodes.

### Ancestral state reconstruction

**Fig. 6:**
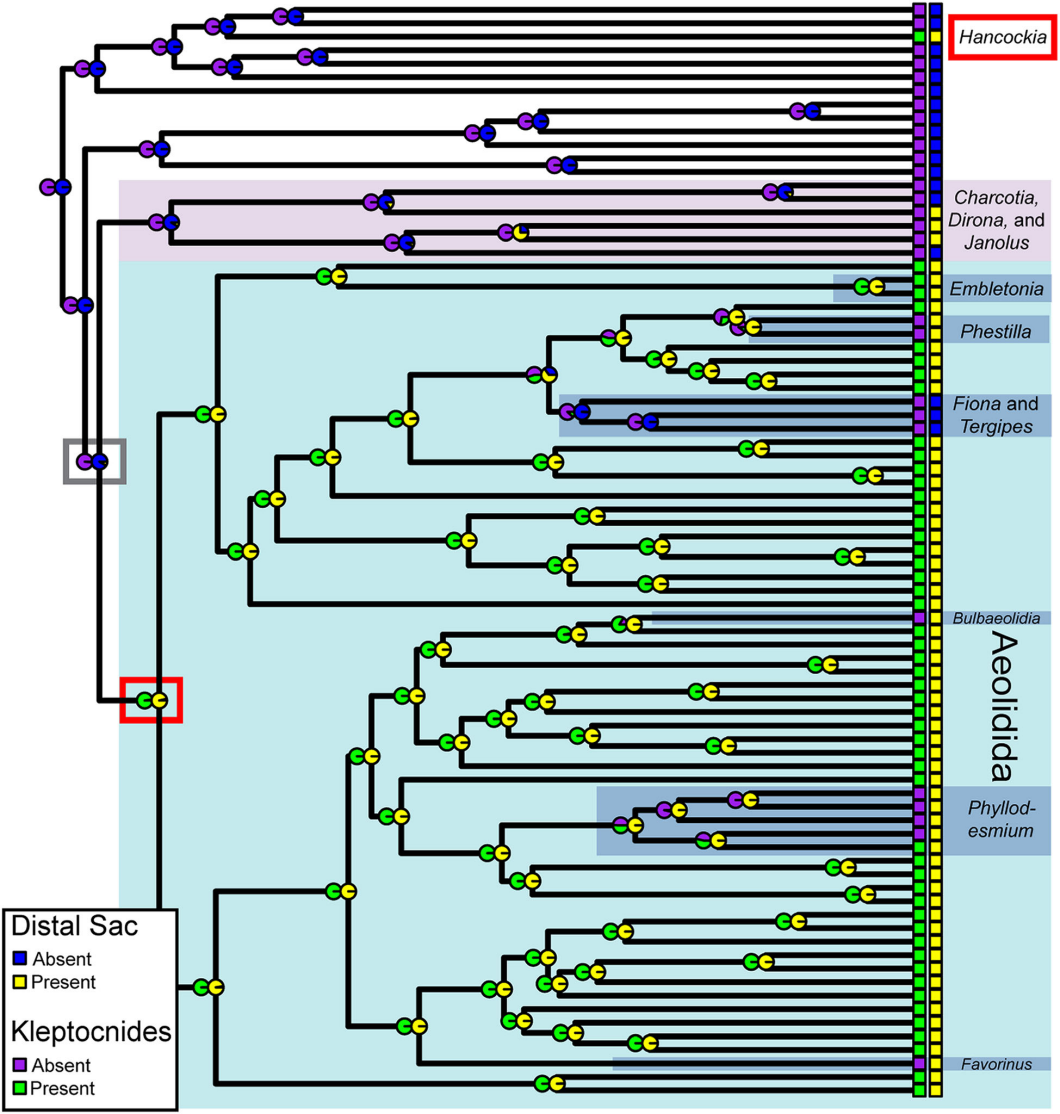
Ancestral state reconstruction analysis for the presence of kleptocnides and a distal sac branching off of the digestive gland. Pie charts on the nodes are scaled marginal likelihoods calculated using the ace function in APE. The red boxes indicate: the node at the base of Aeolidida and the genus *Hancockia*, the two groups in which nematocyst sequestration evolved. The grey box indicates the base of the clade containing Aeolidida and *Charcotia*, *Dirona*, and *Janolus*, which may be where distal sacs originated*.* Also highlighted with blue background boxes are additional genera within Aeolidida where losses and/or re-gains may have occurred

Ancestral state reconstruction supports the hypothesis that the sequestration of nematocysts has originated twice within Cladobranchia, once at the base of Aeolidida and once in *Hancockia* (Fig. 6, red boxes). The presence of a sac at the distal edge of the digestive gland also seems to have originated at least twice (but up to four times). The most likely scenario based on the ancestral state reconstruction is one origin in *Hancockia*, two origins within the Charcotiidae + Dironidae + Proctonotidae clade, and one at the base of Aeolidida. The results also indicate that loss of nematocyst sequestration has occurred four times (in *Phyllodesmium*, Fionidae, *Favorinus*, and *Bulbaeolidia*) and a distal sac has been lost at least once within Aeolidida (in a group of taxa in Fionidae).

## Discussion

### The cnidosac in Cladobranchia

#### Comparative anatomy of the cnidosac

Prior to the present study, numerous assumptions have been made about the uniformity of cnidosac morphology among cladobranchs that harbor kleptocnides [3, 23, 31]. Edmunds [31] in particular provides drawings that are remarkably consistent across species of Fionidae, Favorinidae, Facelinidae and Aeolidiidae, although cnidosac descriptions were not the primary purpose of that publication. All of the species illustrated in that work possess a clear entrance connecting the digestive gland with the cnidosac (though in some cases a slight elongation of the entrance is depicted, similar to a channel) and a distinct exit at the tip of the ceras connecting the cnidosac to the exterior. Additionally, all cnidosacs are depicted to possess kleptocnides, and the way the musculature is presented is also very uniform. The most detailed study on aeolid cnidosacs, until now, was written by Kälker and Schmekel [69], but it did not sufficiently describe the variation that can be found in this structure across the roughly 600 species of aeolids [4]. In addition, work on the cnidosac-like structures in *Hancockia* spp. began only recently [26, 27].

In this study, we find that the length, size and structure of the entrance to the cnidosac varies more than expected based on previous work, as does the structure of the exit, or cnidopore, the musculature surrounding the cnidosac, and the position and orientation of the kleptocnides [3, 29–31, 64, 66, 69, 71]. It is important to note that although we provide broad taxon sampling of cnidosac morphological characters, for many species only one specimen was available for analysis. As such, the observations of absence in certain cases should be taken with caution. We discuss those taxa in particular below.

Previous work presents only a short and simple entrance to the cnidosac (i.e., a direct opening) [30, 31, 66], likely due to the selection of taxa that possess this condition simply by chance. Our work suggests that this is the most common transition between the digestive gland and the cnidosac. However, Hancock and Embleton [64] mention the presence of a ciliated channel in *Aeolidia* (= *Eolis*) *papillosa* and Herdman and Clubb [66] note the presence of a “long, curved connecting duct” in what is now *Facelina bostoniensis* (= *Facelina drummondi)*. A few taxa possess a ciliated channel, including *Aeolidia papillosa*, *Cerberilla amboinensis*, *Cratena peregrina*, *Pteraeolidia ianthina*, and *Paraflabellina ischitana* [formerly *Flabellina*]. These taxa are not closely related, and therefore the channel is not homologous among the taxa that possess it, suggesting a functional explanation for its presence. We initially suspected that the presence of this elongate channel was related to the size of the kleptocnides, as *A. papillosa*, *C. amboinensis*, and *P. ianthina* all sequester larger kleptocnides (> 20 μm in length). However, this is not supported by *C. peregrina* and *P. ischitana*, as these species sequester smaller nematocysts. In these cases, the ciliated channel may be a relict of an ancestral shift in diet, but the possible functional significance of the ciliated channel remains speculative.

Similarly, there is no consistent pattern amongst the taxa that possess a proliferation zone versus those that do not. The one exception is the absence of a proliferation zone in taxa that do not sequester nematocysts from the genus *Phyllodesmium*. The only species within *Phyllodesmium* in which we identified a proliferation zone is *P. jakobsenae*, which is the only species of *Phyllodesmium* known to harbor kleptocnides. It is still unclear why some taxa seem to have a proliferation zone while others do not, but we suspect that in a small number of cases artifacts of the sectioning of some samples have led to the destruction of this region (likely due to difficulties in preservation), which leads to membrane fragments and free-floating kleptocnides within the cnidosacs of some species a (e.g., *Cratena peregrina*; Fig. 4d). To address this point, more individuals from these species should be investigated. The apparent absence of a proliferation zone may also be due to differences in the growth stage of the individuals investigated, or of the cerata if any were in the process of regeneration, but we found no evidence that explicitly supports either of these hypotheses. Further, at least one previous study mentioned the presence of this region [29], but it was not discussed in detail. This region is where nematocysts are taken up by the cnidophages before they migrate towards the distal end of the cnidosac. However, the precise extent of the proliferation zone remains unclear. In some species, it appears to be restricted to the cnidosac (e.g., *Pteraeolidia ianthina*; Fig. 4a), but in others this zone seems to extend into the adjacent parts of the digestive gland (e.g., *Dondice occidentalis*; Fig. 4b). In the majority of taxa that sequester nematocysts, we found only very simple exits from the cnidosac, which in some cases is covered by a thin epithelium. This covering contains cells similar to that of the epidermis of the cerata, which is composed of elongated columnar cells with what appear to be many specialized vacuoles. Simple exits are the most common, both in our study and seemingly in others [31, 66]. However, in a few select taxa within Aeolidiidae, including *Aeolidia papillosa*, *Anteaeolidiella chromosoma*, and *Cerberilla amboinensis*, a complex cnidopore is present (Fig. 4c). It bears an epithelial lining that appears continuous with that of the epidermis. This structure has been identified before [69], but was simply considered a zone of undifferentiated cells that was believed to serve as a reserve for lost cnidophages. However, due to the location at the distal end of the cnidosac, and as part of the cnidopore, we suspect that this is not the case. Instead, we hypothesize that this cell layer is a special adaptation for releasing the exceptionally long and narrow nematocysts sequestered from anemones (up to 50—60 μm in length, but < 5 μm in width). The term cnidopore has previously been used uncritically to refer to all exits from the cnidosac [30, 31], but we now redefine the term cnidopore here to refer to the structure thus far only found in Aeolidiidae.

Although the musculature surrounding the cnidosac also varies across Aeolidida, the significance of this variation is unclear. Musculature around the cnidosac is very thin or lacking entirely in only a few species, including *Embletonia gracilis, Embletonia pulchra* and *Bulbaeolidia alba*. When present, muscle thickness varies across species, ranging from what appear to be one to multiple layers. This variation in the thickness of the musculature is illustrated in one previous study [31], though not as precisely as we indicate here (Table 3). There is no obvious evolutionary explanation for the variation in muscle thickness or number of muscle layers across taxa [72, 73], but a thicker muscle layer would likely result in more forceful expulsion of the kleptocnides. Increased musculature might be associated with predation pressure, size of incorporated kleptocnides, or developmental stage. Incorporating additional individuals from each species, and at different stages of development, as well as measurements of kleptocnide size and muscle thickness would be beneficial for assessing these hypotheses.

The differentiation of cnidophages from a functional, active cell into a “container or larder” of kleptocnides at the tip of the cnidosac reflects the maturation of the kleptocnides via proton transport, which are immature and non-functional when first sequestered [28]. After maturation, the cells appear to have no further functioning due to the reduction of cell complexity. Previous workers have attempted to address the origin of the membrane of the cnidophage [3, 74], and recently have concluded that it is a phagosome, a specialized vesicle formed by the cell membrane [3]. Within cnidophages, the number of kleptocnides may vary both within and among taxa. This appears to be associated with the size of the kleptocnides; there tend to be fewer large kleptocnides (> 20 μm in length) within a given cnidophage compared to those with smaller kleptocnides (usually 10 μm or less). An example can be found in *Pteraeolidia ianthina* (Fig. 4a), which sequesters nematocysts of multiple size classes.

#### Divergences from the general theme

The morphological characters assessed in this study appear to be quite variable within families, but most cnidosacs generally vary on a theme that is conserved across Aeolidida. However, there are still others that have lost particular cnidosac structures or have lost the cnidosac altogether. One might hypothesize that the cnidosacs lose the connection to the digestive gland or the musculature surrounding the cnidosac when no nematocysts are sequestered. For example, species from the genus *Phyllodesmium* (except for *P. jakobsenae*) possess muscle bound cnidosacs that appear to be devoid of kleptocnides, but there are no obvious entrances to the digestive diverticulum or exits to the external environment. Rather, these species sequester chemicals for defense [75], and thus do not necessarily require a structured entrance. In this way, the cnidosacs in *Phyllodesmium* may be similar to the mantle dermal formations in Charcotiidae, which lack an exit but release contents when compressed [41, 76]. However, in species from the genus *Favorinus*, the overall structure of the cnidosac (including the opening from the digestive gland and muscles around the cnidosac) remains the same, but no kleptocnides are present due to the penchant of these species for feeding on the eggs of other gastropods [33]. Although it is possible that the lack of kleptocnides may stem from a hypothetical proclivity in *Phyllodesmium* and *Favorinus* to discharging nematocysts during the fixation process, we suggest this is unlikely. For one, members of *Phyllodesmium* appear to have an intact epithelium where one might have expected the kleptocnides to be ejected. Were kleptocnides ejected during fixation, the ceras would be fixed with an opening in the tip. In addition, the absence of kleptocnides in *Phyllodesmium* in particular is also well documented (with the exception of *P. jakobsenae*) [10, 75, 77–79]. In *Favorinus*, it is possible that this is the case, given the open epithelium at the tip of the cerata (e.g., Fig. 2c), but the absence of kleptocnides is consistent with the habit of members of this genus eating gastropod eggs.

Even more variations on this theme are found in *Bulbaeolidia alba*, *Embletonia* spp., and species within the genus *Fiona*. *Bulbaeolidia alba* has a sac at the distal end of the digestive gland that contains only occasional zooxanthellae (*Symbiodinium*). In addition, we could find no obvious entrance or exit to or from the sac, and the structure appears to be surrounded by a few thin muscle fibers. We hypothesized that the lack of kleptocnides may be due to the very small size of *B. alba*, which might therefore possess a lower defense requirement, but even smaller taxa within the genera *Embletonia* [26] and *Pseudovermis* [80] possess kleptocnides. Alternatively, we hypothesize that a lack of sequestration may be related to the size or utility of the nematocysts found within the anemones on which this species feeds [33]. A third alternative is that *B. alba* instead houses other natural compounds within this sac, either from its prey as in *Phyllodesmium* [79] or produced de novo. Again, it is possible that the lack of kleptocnides in *B. alba* is an artifact of a small sample size, but like *Phyllodesmium,* no evidence of extrusion could be found. Members of Embletoniidae appear to have evolutionarily lost the musculature surrounding the cnidosac entirely or represent an intermediate step in the evolution of the cnidosac, as discussed in the cnidosac evolution section below. There is also no obvious entrance or exit to and from the cnidosac in these taxa. Finally, some species of Fionidae within the genus *Fiona* (this study) have lost the cnidosacs entirely, ostensibly because species in this genus prefer non-cnidarian prey [33].

Sequestered nematocysts have also been found in one other family within Cladobranchia, Hancockiidae. We see structures in *Hancockia californica* that are very similar to cnidosacs (which we call cnidosac-like), with kleptocnides housed in cnidophage-like cells in multiple muscular sacs at the tip of each ceras. These structures have also been found in *Hancockia uncinata* and *H. schoeferti* [27], and in some cases cnidosac-like structures were found in both the cerata and the rhinophoral sheaths. Homology inferences regarding the structures found in Hancockiidae and those in Aeolidida are discussed in the next section.

### Phylogeny of Cladobranchia and evolution of the cnidosac

#### Phylogenetic inferences

Given that much of the molecular data included here are derived from previously published studies, the topology inferred in our phylogenetic analysis (Figure 5) is consistent with that found in both recent phylogenomic studies [19, 81]. However, this work extends previous findings by including taxa not analyzed in recent phylogenomic studies, namely taxa from the genera *Phyllodesmium*, *Caloria*, *Pruvotfolia*, *Pteraeolidia*, *Cratena*, *Facelina*, *Glaucus*, *Calmella*, *Piseinotecus*, *Tergipes*, *Notaeolidia*, *Embletonia*, and *Charcotia*. The majority of these fall within the clades we would expect based on prior molecular work: *Phyllodesmium* is closely related to *Dondice* within the facelinid clade that is sister to Aeolidiidae [82, 83]; *Caloria* is supported within the second facelinid clade and is closely related to species of *Pruvotfolia* [84]; *Facelina*, *Glaucus* and *Cratena* are closely related within the second facelinid clade [83]; *Calmella* is closely related to *Flabellina* and *Paraflabellina* [85, 86]; and *Tergipes* falls within what is now Fionidae [87]. However, the placement of *Pteraeolidia* as closely related to *Palisa* and *Austraeolis* within the second facelinid clade is novel to this study, and the molecular data presented here support the position of *Charcotia* within the sister group to Aeolidida, as suggested previously by morphological work [15].

Despite the addition of all of the new data presented here, the positions of *Notaeolidia* and *Embletonia* remain unclear [26]. Support for the exact positions of these two genera is poor, and these taxa appear to contribute to the low bootstrap values at the base of Aeolidida. This may be due to long-branch attraction between *Notaeolidia* and *Flabellinopsis iodinea* [formerly *Flabellina*], and between *Embletonia* and *Unidentia*. However, morphological analyses also support at least the earlier divergence of *Notaeolidia* within Aeolidida [15, 88]. The uncertainty surrounding the affinities of these four taxa has implications for our understanding of the evolution of the cnidosac.

We also find that individual gene tree analyses (and the three genes analysis) are consistent with previous large-scale PCR-based sequence analyses [21]. These topologies and posterior probabilities support the idea that PCR-based sequencing data for the genes used (COI, 16S, and 18S) provide some utility for inferring recent divergences, but that high-throughput sequencing data are necessary for inferring deeper divergences.

#### Cnidosac evolution

The sequestration of cnidarian nematocysts has originated at least twice within Cladobranchia based on the phylogeny presented here (Fig. 6). This result also indicates that species within Aeolidida that do not sequester nematocysts have lost this ability, which seems to have occurred at least three times. In addition, the early divergence of Embletoniidae within the aeolid phylogeny is suggestive; it indicates that the structure within Embletoniidae is a cnidosac, and that the lack of musculature around the cnidosac may represent an intermediate step in the evolution of kleptocnide sequestration. However, stronger support for relationships at the base of Aeolidida are necessary before further inferences can be made. Our results also support several independent losses of the cnidosac, including in members of *Fiona* and *Tergipes*. This appears to be due to a switch to preying mostly on Crustacea in *Fiona* [33]. A prey preference transition from hydroids to other types of organisms may also have led to the loss of cnidosacs in some species of *Tergipes* [33, 89]*.*

The presence of a sac at the distal end of the digestive gland is hypothesized to have originated prior to the ability to sequester nematocysts (Figure 6; grey box), although this result relies on the hypothesis that the terminal sacs found in Charcotiidae and Proctonotidae [41, 43, 76, 90] are homologous to those in Aeolidida. Support for this hypothesis is very low, and thus appears unlikely based on our reconstruction. However, the terminal sacs of Charcotiidae and Proctonotidae are considered to function as excretory structures, and some have hypothesized that the aeolid cnidosac is an adaptation of this sac for defense [76]. Although the homology remains uncertain, our ancestral state reconstruction does not completely reject this modification hypothesis, wherein the distal sac was exapted to sequester nematocysts. More morphological and molecular data from additional species in the Charcotiidae + Proctonotidae + Dironidae clade is necessary to further test this hypothesis, as this clade is not well represented in our analysis, which can hinder evolutionary inferences.

The cnidosac-like structures in Hancockiidae [26, 27] appear to have evolved independently from the distal sac in both Aeolidida and its sister clade. This is supported by the phylogenetic analyses presented here as well as differences in the sequestration process between *Hancockia* and species of aeolids. For example, it appears that *Hancockia* species encapsulate nematocysts in the lumen of the digestive tract before transport [27], unlike aeolids.

## Conclusions

Here, we describe the morphology of the cnidosac and cnidosac-like structures across all major clades of Cladobranchia in which it has been identified, and discuss possible functions for variation in structural characters. Overall, we find that cnidosac morphological characters are variable across Cladobranchia, and we provide evolutionary hypotheses in many cases that might explain the evolutionary patterns found. We also conclude that the sequestration of nematocysts has originated at least twice within Cladobranchia and that the sac at the distal end of the digestive gland may have originated prior to that of the sequestration of nematocysts. Finally, support for the origin of a distal sac prior to that of nematocyst sequestration suggests that the terminal sacs found in Charcotiidae and Proctonotidae may be homologous to the cnidosacs found in Aeolidida. Taken together, this research provides a more thorough understanding of the evolution of morphological characters relating to nematocyst sequestration in Cladobranchia.

## Additional files

Additional file 1: Specimen information for the morphological data used in this study. (XLSX 10 kb)

Additional file 2: Specimen information for the molecular data analyzed in this study. (XLSX 20 kb)

Additional file 3: Primers used for fragments of CO1, 16S and 18S. (XLSX 37 kb)

Additional file 4: Polymerase chain reaction (PCR) cycling protocols for each of the three genes. (XLSX 40 kb)

Additional file 5: Convergence statistics and plots for the COI Bayesian analysis. (PDF 1966 kb)

Additional file 6: Convergence statistics and plots for the 16S Bayesian analysis. (PDF 2004 kb)

Additional file 7: Convergence statistics and plots for the 18S Bayesian analysis. (PDF 1646 kb)

Additional file 8: Convergence statistics and plots for the 3 genes Bayesian analysis. (PDF 1564 kb)
