# Supplementary material for: Comparative morphology and evolution of the cnidosac in Cladobranchia (Gastropoda: Heterobranchia: Nudibranchia)
Source: Front Zool. 2018 Nov 13;15:43. doi: 10.1186/s12983-018-0289-2 (PMC6234619; doi:10.1186/s12983-018-0289-2)

16S Convergence Plots

This document contains plots and statistics for evaluating the convergence of 16S MrBayes runs performed in this paper. Assessment of convergence used the web address http://danwarren.net/plot-comparisons.html written by the authors of the package **RWTY**.

It seems that both 16S runs have converged. The approximate sampling Estimated Sample Size (ESS) and topology ESS are both above 200 (standard rule of thumb). The tree topology trace shows well-mixed chains and a clear optimum, cumulative split frequencies seem to have become fairly stable, and sliding window split frequencies show large jumps, but a clear search of the tree space. Both runs also seem to have converged on similar posterior probabilities, as shown by the split frequency comparisons. The average standard deviation of split frequencies (ASDSF) is below 0.01 and shows a consistent decrease, as expected. Tree space plots show both runs searching similar areas of tree space and well-mixed chains. The topological autocorrelation plots also appear to have reached a plateau for each run.

Analysis code

```r
library(rwty)
```

```
## Loading required package: ape

## Loading required package: ggplot2
```

```r
# Pull in the trees (only need to indicate a folder)
my.trees <- load.multi("16S", format='mb')
```

```
## [1] "16S_con_cds_modified.nex.run1.t"
## [1] "Reading trees..."
## [1] "1000 generations per tree..."
## [1] "Trees are unrooted..."
## [1] "Reading parameter values from 16S_con_cds_modified.nex.run1.p"
## [1] "16S_con_cds_modified.nex.run2.t"
## [1] "Reading trees..."
## [1] "1000 generations per tree..."
## [1] "Trees are unrooted..."
## [1] "Reading parameter values from 16S_con_cds_modified.nex.run2.p"
```

```r
# Set burn in
burnin_val <- 2500

# Analysis of trees using **RWTY**
my.trees.rwty <- analyze.rwty(my.trees, burnin=burnin_val, fill.color='LnL')
```

```
## [1] "Creating trace for LnL"
## [1] "Creating trace for LnPr"
## [1] "Creating trace for TL"
## [1] "Creating trace for r.A...C."
## [1] "Creating trace for r.A...G."
## [1] "Creating trace for r.A...T."
## [1] "Creating trace for r.C...G."
## [1] "Creating trace for r.C...T."
## [1] "Creating trace for r.G...T."
## [1] "Creating trace for pi.A."
## [1] "Creating trace for pi.C."
```

```
## [1] "Creating trace for pi.G."
## [1] "Creating trace for pi.T."
## [1] "Creating trace for alpha"
## [1] "Creating trace for m.1."
## [1] "Creating trace for tree topologies"
## [1] "Calculating approximate ESS with sampling intervals from 1 to 100"
## [1] "Creating topological autocorrelation plot"
## [1] "Creating sliding window split frequency plot for 20 clades"
## [1] "Creating sliding window ACSF plot"
## [1] "Creating cumulative split frequency plot for 20 clades"
## [1] "Creating cumulative ACSF plot"
## [1] "Creating treespace plots"
```

```
## Warning: `panel.margin` is deprecated. Please use `panel.spacing` property
## instead
```

```
## Warning: `panel.margin` is deprecated. Please use `panel.spacing` property
## instead
```

```
## [1] "Creating ASDSF plot"
## [1] "Creating split frequency matrix and ASDSF clustering plots"
```

```r
# Approximate ESS for topologies
topological.approx.ess(my.trees,burnin=burnin_val)
```

```
## [1] "Calculating approximate ESS with sampling intervals from 1 to 100"
```

```
##   operator approx.ess                          chain
## 1        =   594.9638 16S_con_cds_modified.nex.run1.t
## 2        =   577.6709 16S_con_cds_modified.nex.run2.t
```

Plots

Estimated Sample Size

```
makeplot.pseudo.ess(my.trees, burnin = 2500)
```

```
## [1] "Creating pseudo ESS plot"
## [1] "Calculating pseudo ESS for 7501 trees and 20 replicates, please be patient"
## [1] "Calculating pseudo ESS for 7501 trees and 20 replicates, please be patient"
```

```
## $pseudo.ess.plot
```

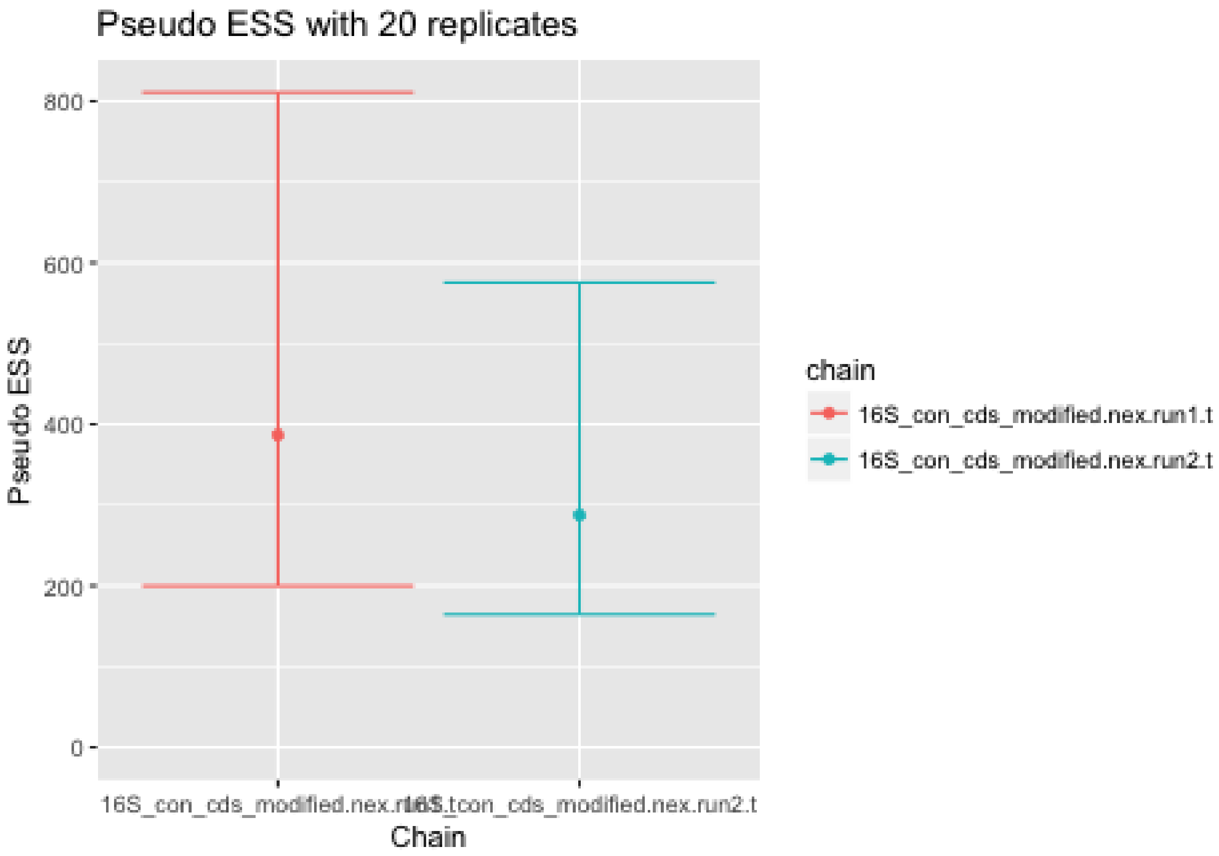

Parameter plot

```
my.trees.rwty$LnL.trace[[1]]
```

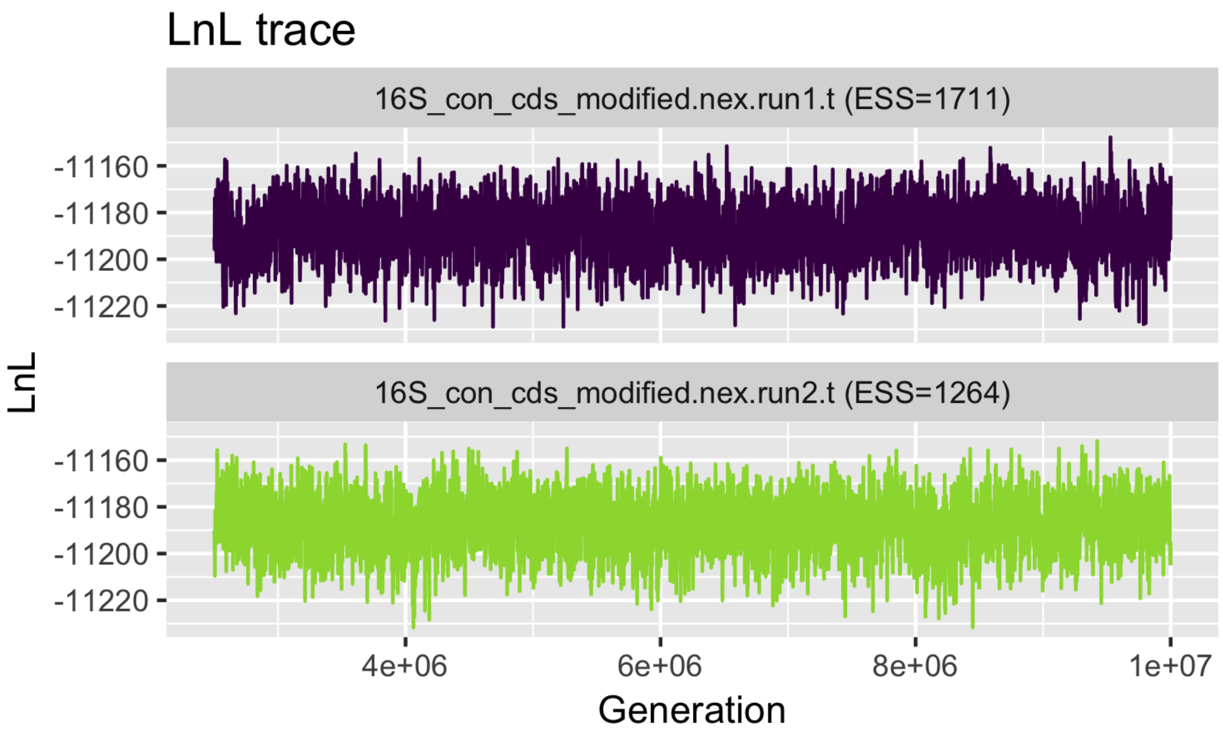


```
my.trees.rwty$LnL.trace[[2]]
```

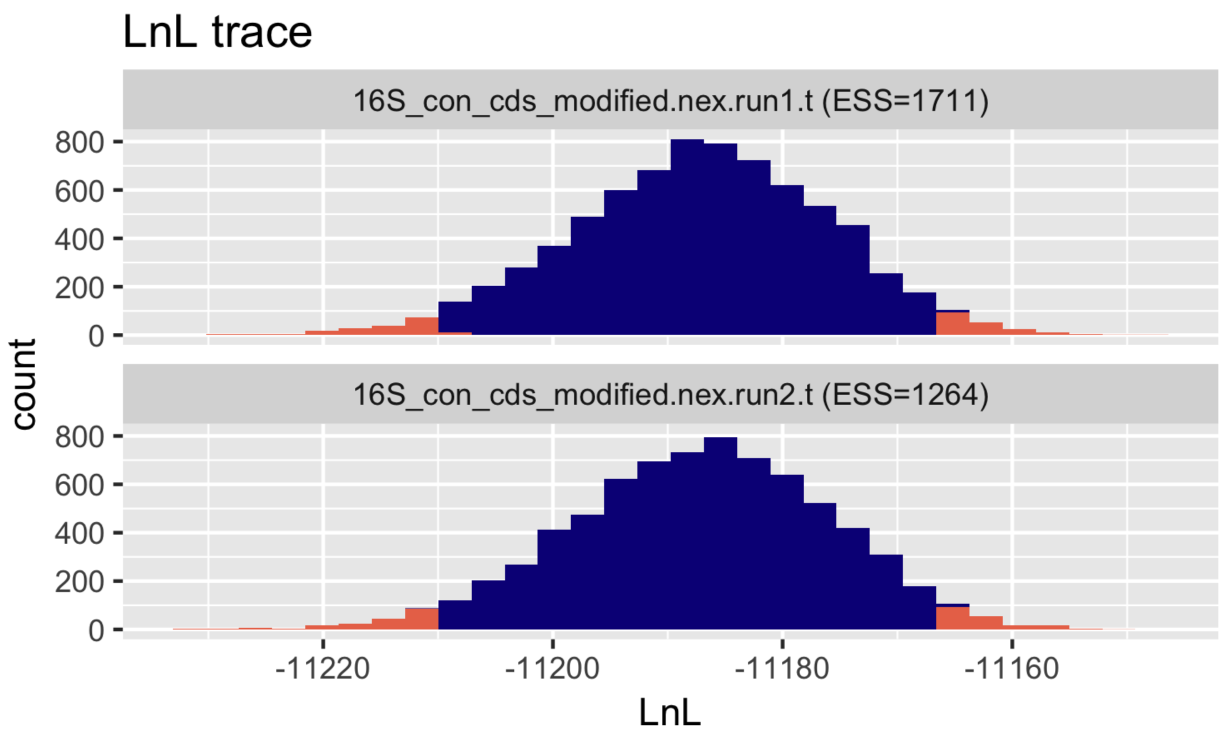

Topology trace plots

```
my.trees.rwty$topology.trace.plot[[1]]
```

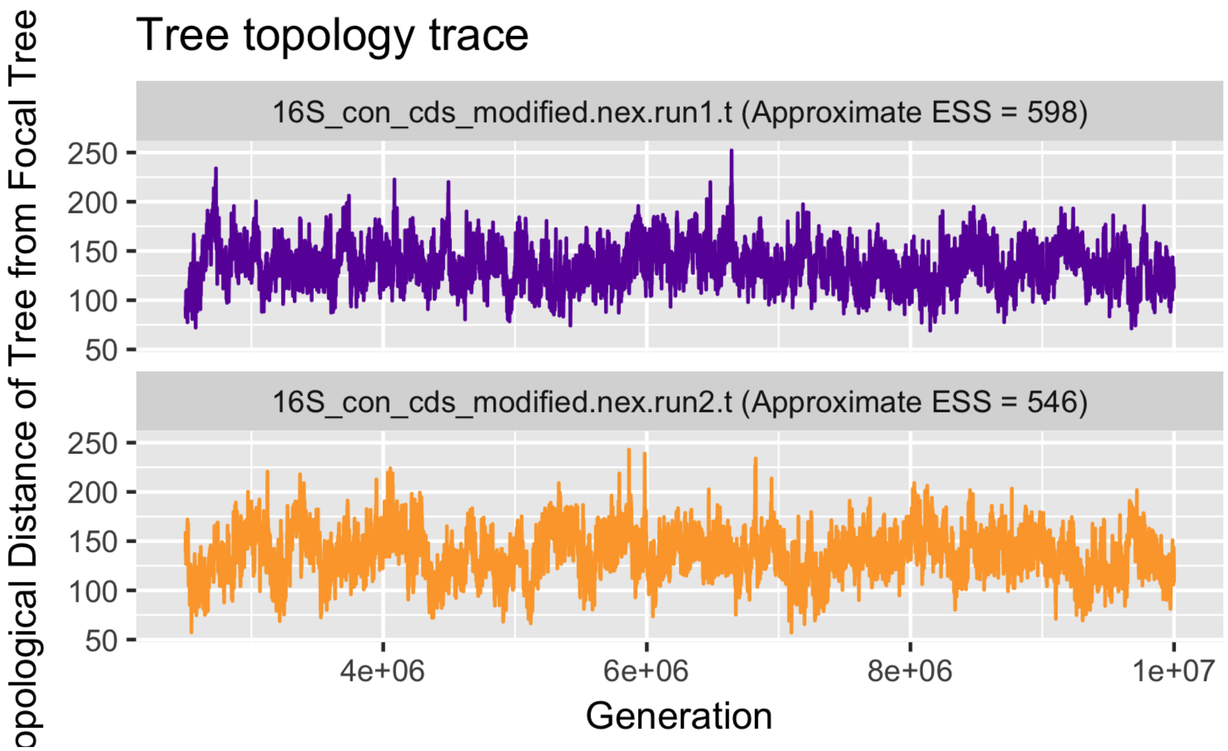


```
my.trees.rwty$topology.trace.plot[[2]]
```

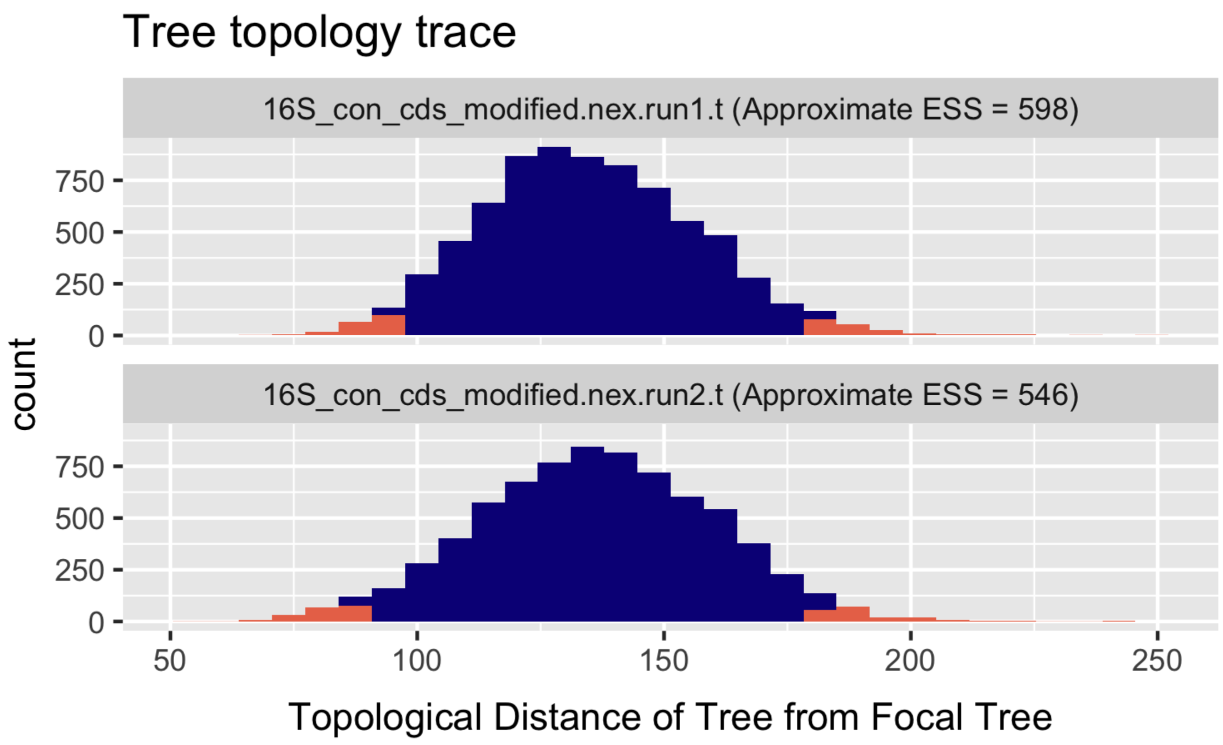

Split frequency plots

```
my.trees.rwty$splitfreqs.cumulative.plot
```

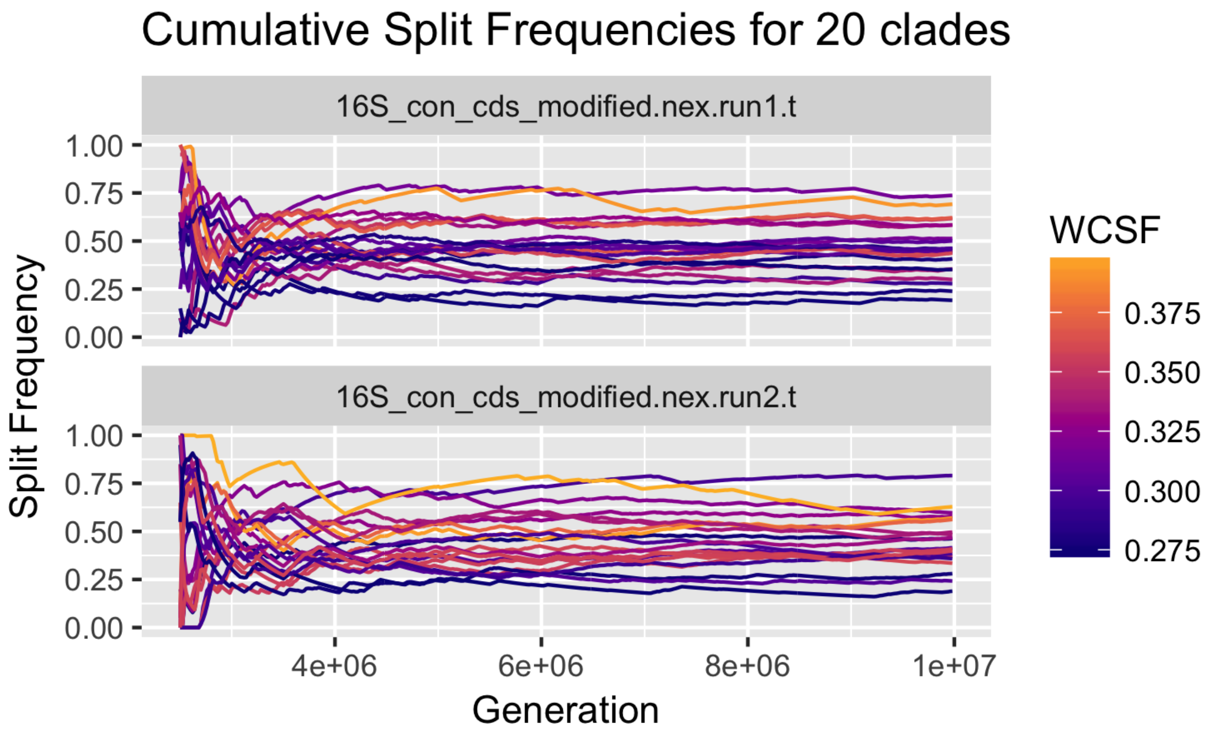


```
my.trees.rwty$splitfreqs.sliding.plot
```

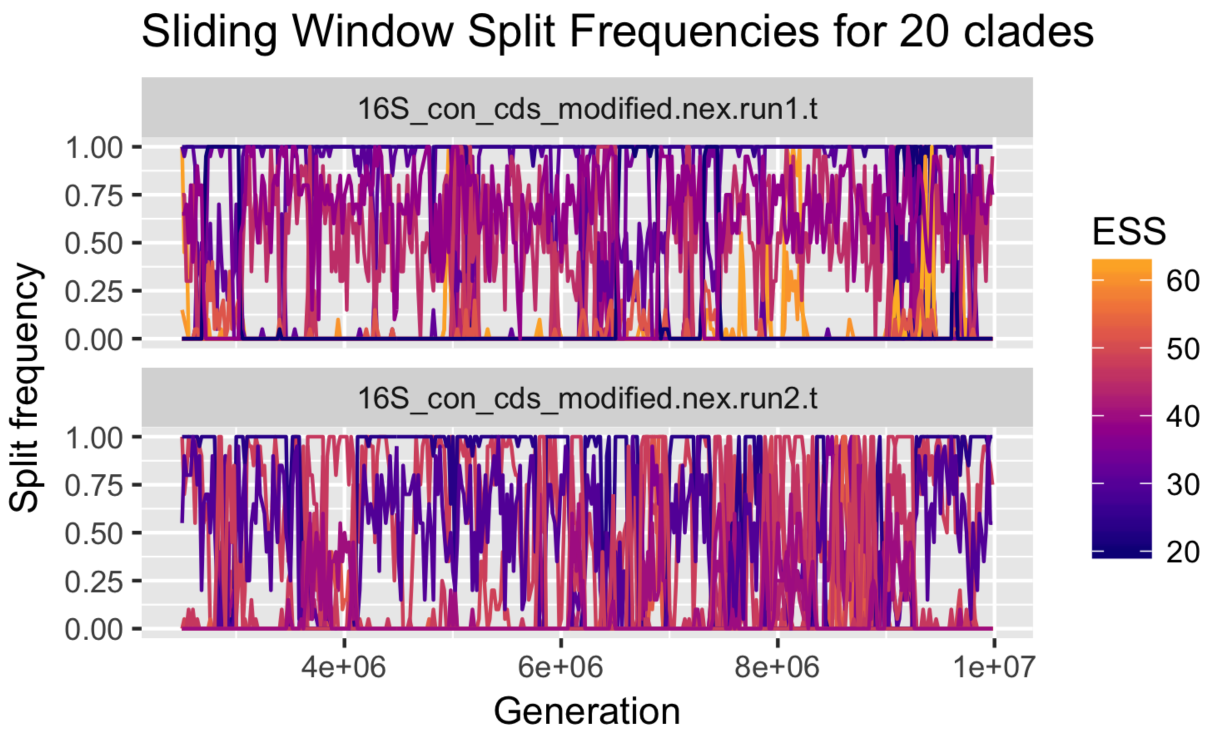

```
my.trees.rwty$splitfreq.matrix
```

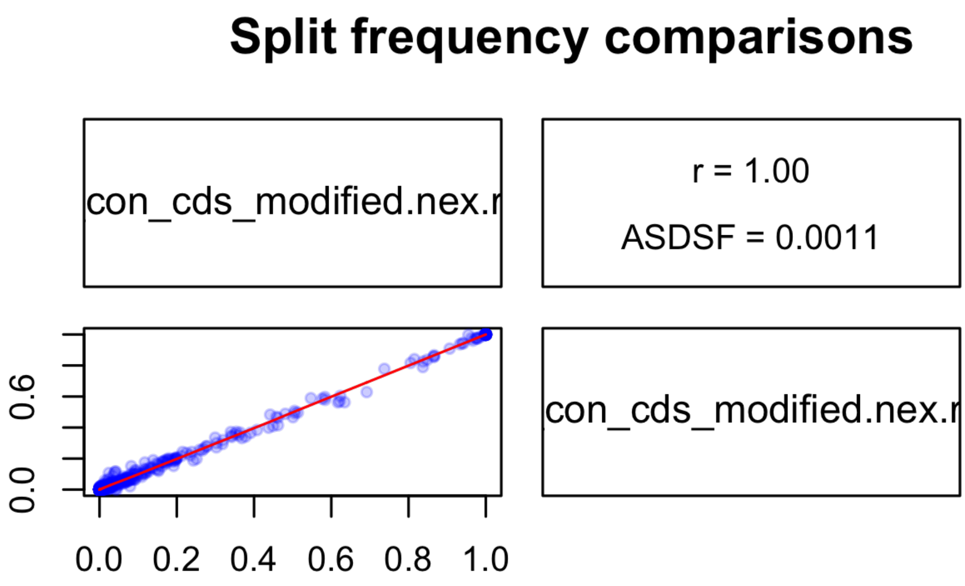


```
my.trees.rwty$asdsf.plot
```

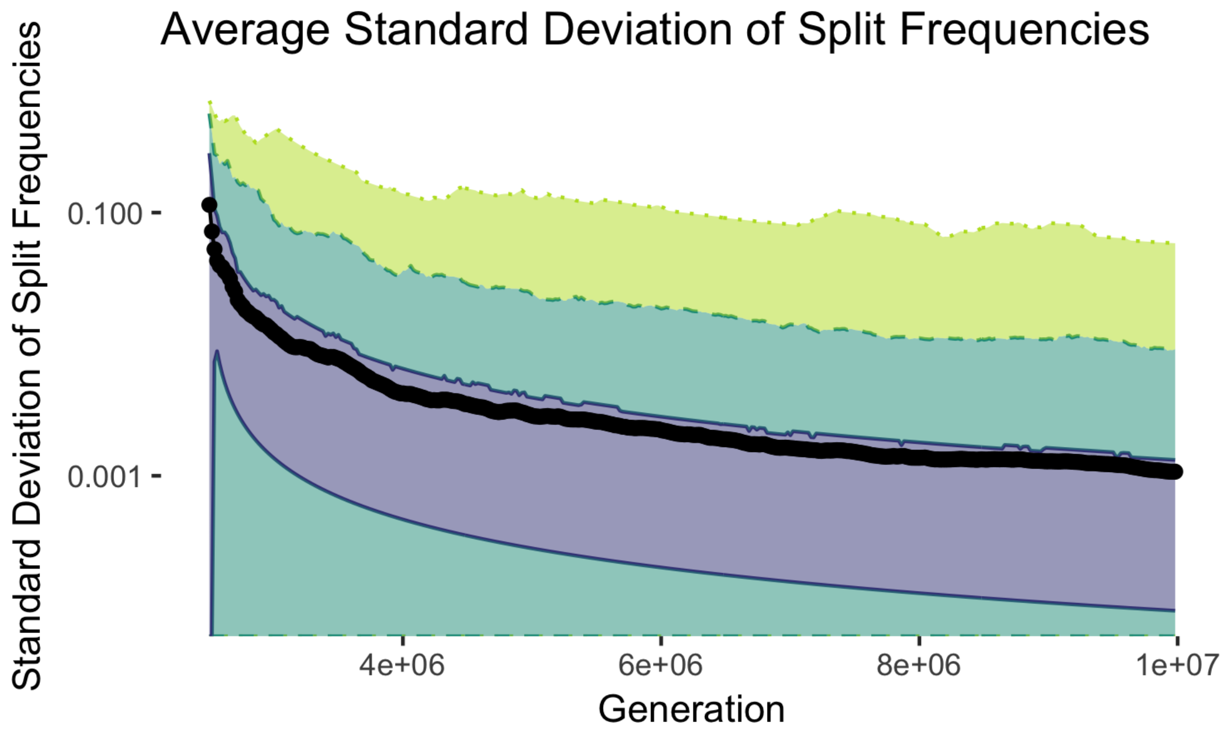

Tree space plots

```
my.trees.rwty$treespace.heatmap
```

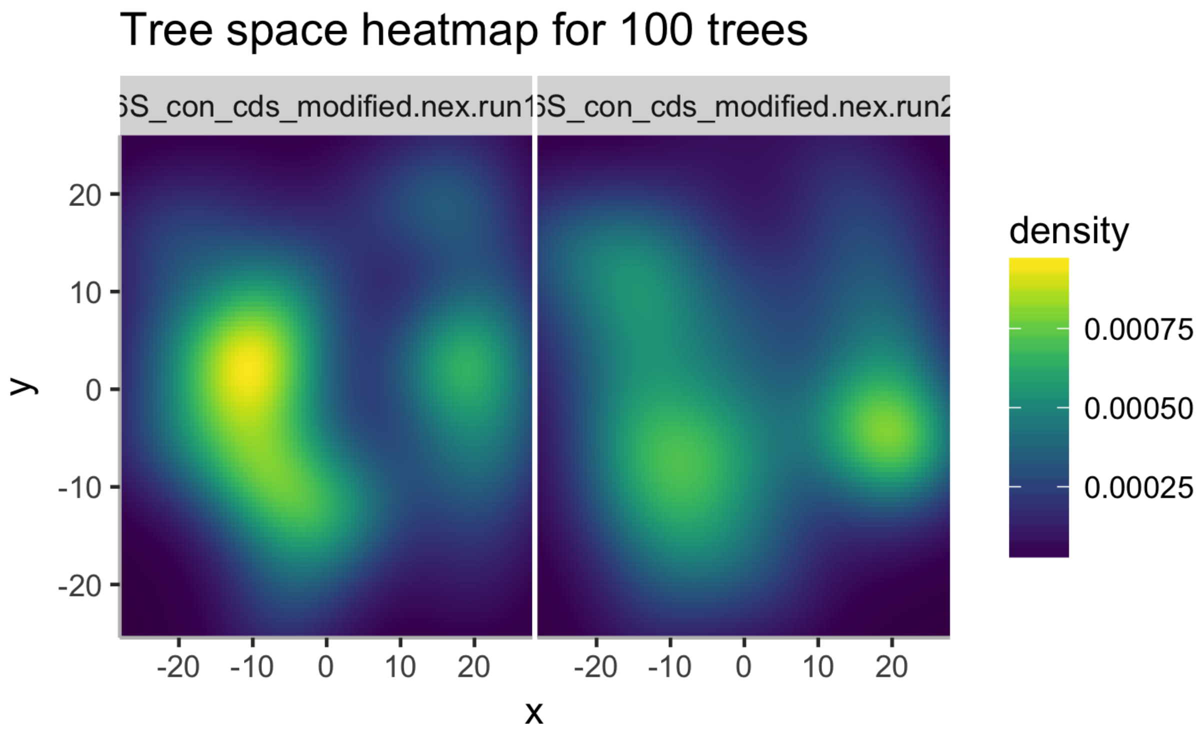


```
my.trees.rwty$treespace.points.plot
```

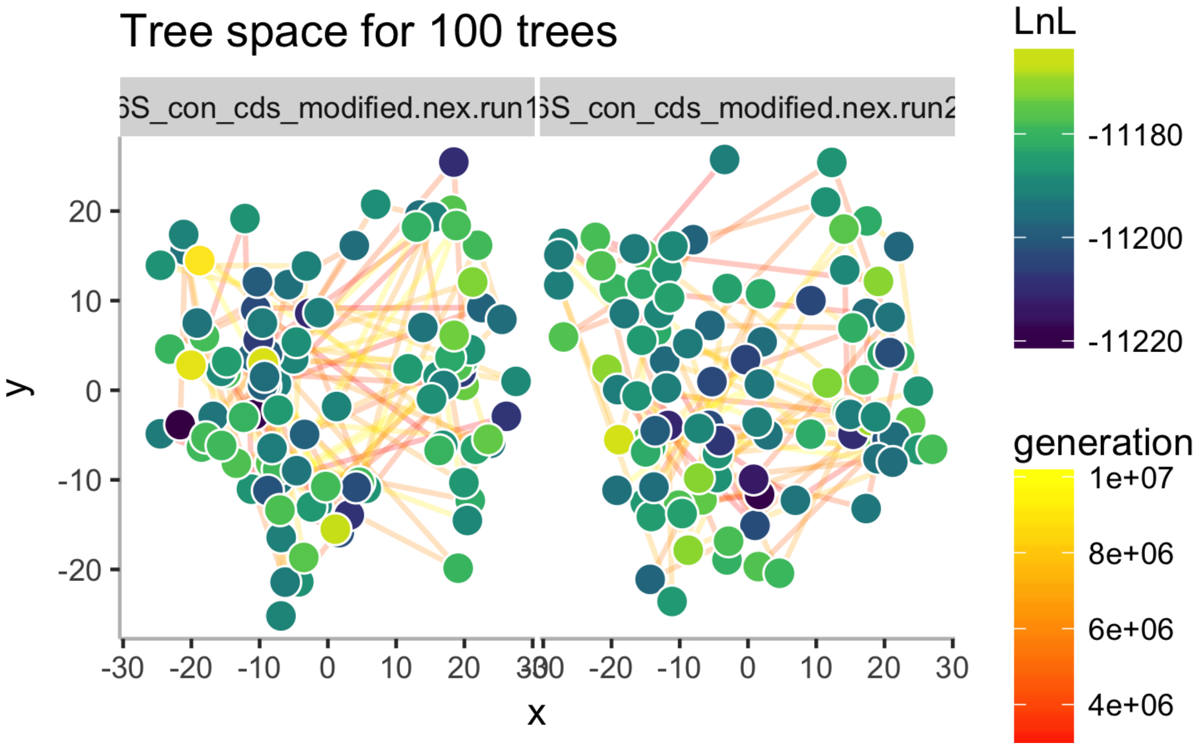

Autocorrelation plots

```
my.trees.rwty$autocorr.plot
```

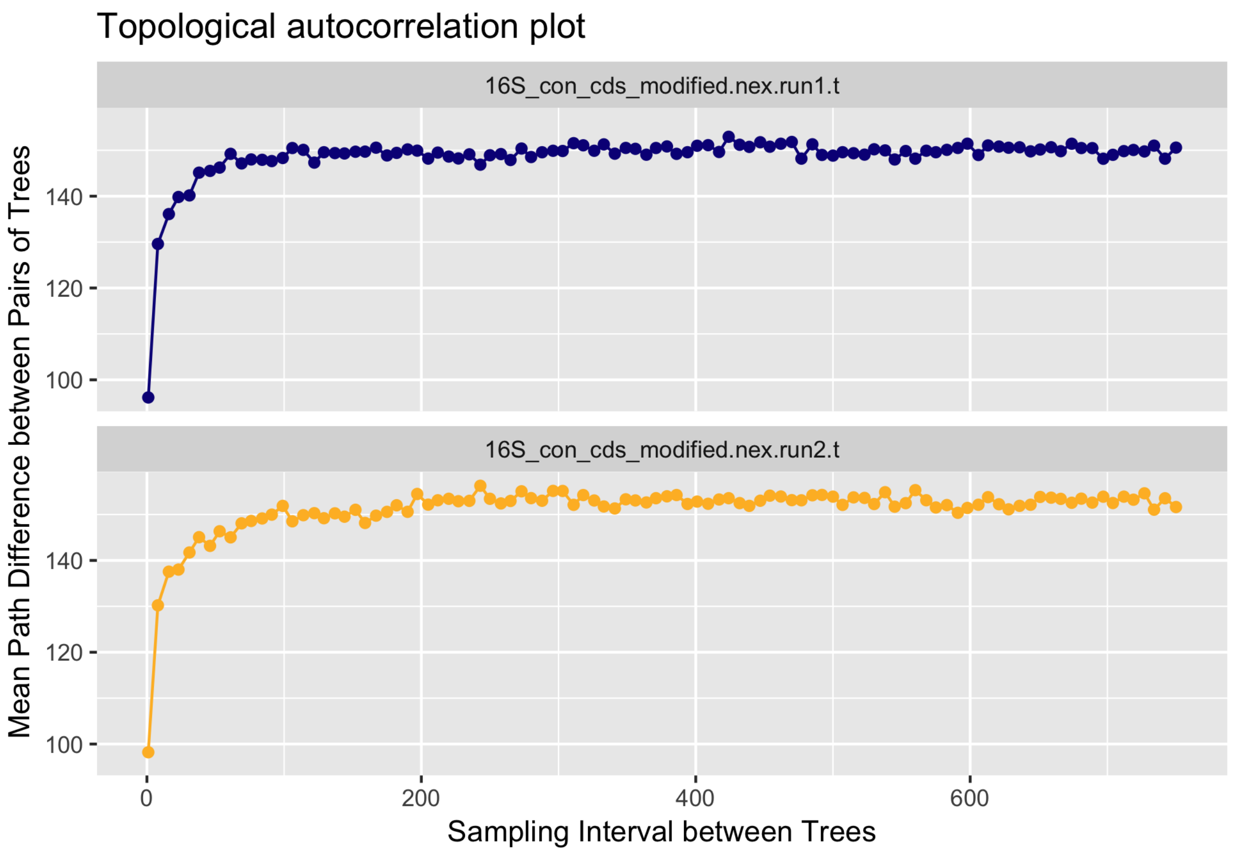

Supplement: Supplementary file 6 — Convergence statistics and plots for the 16S Bayesian analysis. (PDF 2004 kb) [file 12983_2018_289_MOESM6_ESM.pdf]
